# Restricted cubic splines for modelling periodic data

**DOI:** 10.1371/journal.pone.0241364

**Published:** 2020-10-28

**Authors:** Lara Lusa, Črt Ahlin

**Affiliations:** 1 Faculty of Mathematics, Natural Sciences and Information Technologies, University of Primorska, Koper/Capodistria, Slovenia; 2 Institute for Biostatistics and Medical Informatics, Medical Faculty, University of Ljubljana, Ljubljana, Slovenia; 3 Doctoral student of Statistics, University of Ljubljana, Ljubljana, Slovenia; Georgia State University, UNITED STATES

## Abstract

In regression modelling the non-linear relationships between explanatory variables and outcome are often effectively modelled using restricted cubic splines (RCS). We focus on situations where the values of the outcome change periodically over time and we define an extension of RCS that considers periodicity by introducing numerical constraints. Practical examples include the estimation of seasonal variations, a common aim in virological research, or the study of hormonal fluctuations within menstrual cycle. Using real and simulated data with binary outcomes we show that periodic RCS can perform better than other methods proposed for periodic data. They greatly reduce the variability of the estimates obtained at the extremes of the period compared to cubic spline methods and require the estimation of fewer parameters; cosinor models perform similarly to the best cubic spline model and their estimates are generally less variable, but only if an appropriate number of harmonics is used. Periodic RCS provide a useful extension of RCS for periodic data when the assumption of equality of the outcome at the beginning and end of the period is scientifically sensible. The implementation of periodic RCS is freely available in peRiodiCS R package and the paper presents examples of their usage for the modelling of the seasonal occurrence of the viruses.

## Introduction

In biomedicine it is increasingly common to record several patients’ characteristics with the aim to evaluate which of these explanatory variables might be associated with the outcome of interest and, subsequently, to use regression models for the analysis [1].

In some studies it is scientifically meaningful that the values of the outcome variables change periodically over time. For example, researchers studying the occurrence of viruses and collecting data over multiple years are interested in estimating their seasonal variation [2–5]; the period in this case is generally the calendar year and the explanatory variable is defined as the day of the year (as a number), aggregating the measurements made over different years. In this way all the years are treated equally and only the information about the day of the measurement, the *season* within the year, is considered. A similar approach can be used for studying seasonal variation of antibiotic prescriptions or of elective admissions to hospitals. Another example is in fertility research, where the researchers are interested in estimating the hormonal fluctuations, the period being the woman’s menstrual cycle and the outcome variable being the hormone levels for all the days within the period [6, 7]. In the modelling of the circadian rhythm of hormones the period is the 24 hour cycle [8].

In this paper we will focus on modelling periodic numerical explanatory variables using regression models, either when the focus is to model their association with the outcome or merely to adjust the regression analysis for the periodic variable. Cubic splines (CS) or restricted cubic splines (RCS) are a popular way to flexibly model non-linear relationships in regression models [9]. Numerous other types of splines exist and those most widely used were recently surveyed [10].

For example, Strle and colleagues [11] used RCS to estimate the shape of the association between the day of the diagnosis of erythema migrans (EM) and its duration.

They found that the patients diagnosed at the beginning and at the end of the year had longer duration, but the estimates obtained at the extremes of the period differed substantially (55 days on January 1 and of 35 days on December 31, with wide confidence intervals at the extremes). This inconsistency was a consequence of using RCS for modelling periodic data, a method that does not make any use of the information about the periodicity of the explanatory variable: it does not constrain the estimates to be equal or similar at the extremes, nor does it constrain the curves to be smooth if the extremes are joined. It is also common to superimpose smooth curves that do not take periodicity into account over histograms that display monthly aggregated over many years [4].

In this paper we will show how to adapt RCS to model periodic explanatory variables. We will derive the spline function imposing additional constraints that take the periodicity into account, requiring the equality and smoothness in the extremes. We will illustrate the use of periodic RCS using examples from virology research [2] and illustrate the use of the peRiodiCS [12] R package [13] that we developed for this purpose. We will also present the use of periodic CS, originally proposed by [6], and of the cosinor models, which use sine and cosine transformations of the periodic explanatory variable and thus constrain the estimates to be periodic. Additionally, we will include a selected set of simulation studies that are helpful for illustrating the properties of the investigated methods.

## Materials and methods

### Periodic RCS

We present the derivation of a periodic RCS that can be used to model periodic explanatory numerical variables. To illustrate our idea we briefly introduce CS and RCS, and explain the additional constraints used to take the periodicity into account. The technical details and the formulas are given in the S1 Appendix.

#### A brief non-mathematical introduction to CS and RCS

Splines are generally used in regression models when the assumption of linearity between an explanatory variable and (some transformation) of the outcome is not satisfied. To overcome the problem, splines transform the explanatory variable by splitting the range of values in intervals and fitting a separate curve in each interval. The spline is defined so that the overall resulting curve is smooth and continuous. The points that delimit the intervals are called knots.

CS use cubic polynomials, joined so as to obtain a smooth function, imposing the equality of the first and second derivatives in each knot. Using *k* knots the numerical values of the explanatory variables are transformed into *k* + 3 new variables (a linear, a quadratic, a cubic term and other truncated cubic polynomials, see the S1 Appendix for details), known as the basis functions; each basis function is linear in the regression coefficient and, therefore, the coefficients can be estimated using standard methods for multivariable regression model fitting.

RCS are defined as CS, but use linear functions beyond boundary knots (before the first and after the last knot), improving the behaviour at the extremes [14]. The simpler functional form at the extremes implies that only *k* − 1 regression parameters need to be estimated.

The obtained regression coefficients determine the shape of the estimated spline function and usually are not interpreted. Standard statistical inference methods can be used to derive the confidence intervals of the estimated curve or to assess if there is an overall association between the explanatory variable and the outcome (testing if all the spline regression coefficients are equal to zero), or if the association is linear (comparing the nested models that contain only the linear term or the complete spline). To overcome the difficulties in the interpretation of the spline regression coefficients it is customary to present the results graphically, displaying the estimated shape of the spline function with its confidence intervals, or to choose some values of the explanatory variable and evaluate the estimated outcome at these values.

Usually, the position of the knots does not have a major impact on the results and the choice of equally spaced quantiles is recommended [1]. In our analyses we used the default knot positions from the rms R package [15]. The number of knots has an impact on the results, for RCS it is suggested that 5 or less knots are appropriate in most applications and that a data-based optimization of the number of knots can be based on the Akaike information criteria (AIC) [1]. AIC is a measure based on the goodness of fit of the model that includes a penalty for the number of estimated parameters: the goodness of fit improves using models with more parameters (for example, splines with more knots) and AIC favours simpler models when the goodness of fit is similar.

#### Periodic CS and RCS

In most circumstances it is sensible to assume that the outcome values should be equal at the beginning and at the end of the period. For example, when data from multiple years are aggregated, big discontinuities between December 31 and January 1 should not be sensible in most cases since the days in the calendar year are a convention. Similarly, we should not expect big differences between midnight and a moment after it if the data from multiple days are aggregated.

To take this property of periodic data into account we impose the additional constraint that the value of the spline function is equal at the extremes of the period. Additionally, we require that the splines remain smooth if we join the beginning and the end of the period (i.e., we require that the derivatives at the extremes are equal). These additional constraint further reduce the number of estimated regression parameters. Using *k* knots, *k* − 3 parameters are estimated for periodic RCS (instead of *k* − 1), *k* for periodic CS (instead of *k* + 3). For example, for periodic RCS with 5 knots the explanatory variable is transformed into 2 new variables.

The derivation of the basis functions for the periodic spline functions is given in the S1 Appendix. The interested reader can use the web application that we developed [16] to visualize the shape of the basis functions of RCS, periodic RCS and periodic CS.

It is important to note that the empirical procedure for using periodic splines is the same as for non-periodic splines, despite the additional constraints. In practice, the user specifies the number and position of the knots, derives the basis functions values and can use them as a set of explanatory variables in any regression model. Similarly as for the non-periodic splines, the values of the estimated regression coefficients per se are not meaningful and can be used as described for regular splines. A statistical testing procedure can be used to assess if there is an overall association between the explanatory variable and the outcome, while a test for linearity cannot be readily obtained from a periodic RCS or CS, as an independent linear term is not used.

#### Implementation of periodic RCS and CS in peRiodiCS R package

The peRiodiCS R package [12] calculates the values of the basis functions, which can subsequently be used within any regression formula in the R language. The values of the basis functions can also be exported in text format and be used with a different software. The package includes also the functions for the calculation of the periodic CS proposed by [6], a virology dataset [2] and usage examples. Some examples of how to use the peRiodiCS package are given in the S1 Appendix.

### The cosinor model

We evaluated also the performance of the cosinor model, which maps the time explanatory variable in the (0, 2*π*) interval and uses a sine and a cosine transformation of the transformed time as explanatory variables; this approach was proposed by Halbert et al. [17] to model periodic data. In practice, in our real data examples the week time variable was transformed in $w=2\pi \frac{\left(week-1\right)}{52}$ and the sine and cosine of *w* were used as explanatory variables, requiring the estimation of 2 parameters. This model fits one harmonic sinusoidal regression function that can capture only one local minimum and/or maximum.

Extensions of the cosinor model can capture more general types of periodicity. We estimated also a model that can capture bimodal periodicity, by including two additional explanatory variables (*sin*(2*w*) and *cos*(2*w*)); in the following the model will be referred to as the cosinor(2h) model (cosinor model with two harmonics).

The cosinor models guarantee the equality of the estimates at the beginning and at the end of the period, and have the desirable property of not depending on the choice of the starting point of the period: the models fitted using different starting points obtain exactly the same estimates.

### Metrics used to evaluate the models

In our applications we focused on binary outcomes and used logistic regression models.

We used four metrics to evaluate the overall performance of the estimated models: Brier score, the *c* concordance index, the calibration intercept and slope. A brief description of the measures is given below, details can be found in [18].

Brier score measures the difference between observed and predicted outcomes, it is defined as the mean squared difference between the actual binary outcomes and predicted probabilities and it ranges between 0 (perfect model) and *p* × (1 − *p*)^2^ + (1 − *p*) × *p*^2^ (non-informative model), where *p* is the outcome prevalence. Brier score can be decomposed in discrimination and calibration aspects, which are measured by the other metrics.

The *c* concordance index is equivalent to the area under the ROC curve (AUC) for binary outcomes and it is the proportion of all pairs of observations with opposite outcomes that are correctly ranked by the model. It measures discrimination, i.e., how well a prediction model can discriminate patients with the outcome from those not experiencing it; non-informative models have *c* = 0.5, those with perfect discrimination attain *c* = 1.

Calibration measures the agreement between observed outcomes and predictions and can be evaluated only on independent data. The model estimated on training data is used to obtain the estimated linear predictor of new (test) data, which is used as the only predictor in a logistic regression model for the new outcome; the calibration slope is the obtained regression coefficient of the linear predictor, while the calibration intercept is obtained as the intercept from the model that fixes at unity the regression coefficient of the linear predictor (used as an offset). The perfectly calibrated model has a calibration intercept equal to 0 and calibration slope equal to 1. Deviations from the value of 0 of the calibration intercept indicate deviations between the observed outcome and predictions, i.e., that the model underestimates or overestimates the number of events in the new data; calibration slope measures the average strength of the predictors, values below 1 typically indicate overfitting or systematic differences between the data used to develop the model and the new data.

### Real data analysis

We illustrated the modelling of periodic variables by reanalyzing the data from patients with severe acute respiratory infections (SARI), which were collected in the Eastern Mediterranean Region from November 2007 till January 2014 [2]. The positivity to 7 viruses (respiratory syncytial virus (RSV, sample size *n* = 24503), adenovirus (AdV, *n* = 9402), human metapneumovirus (hMPV, *n* = 9384), human parainfluenza virus types 1,2 and 3 (hPIV1–3, *n* = 9402) and human influenza (INF, *n* = 28438)) was tested by real time reverse transcriptase polymerase chain reaction (rtRT-PCR) or PCR. These viruses are known to follow seasonal patterns [2].

We used RCS, periodic RCS and periodic CS and the cosinor models to estimate the seasonality of the viruses with logistic regression models; each virus positivity was an outcome and the week in the year was the periodic explanatory variable. The number of knots for the splines was selected to minimize the AIC (values between 3 (5 for periodic RCS) and 10 were used, considering at least 3 knots and 2 estimated parameters). The results were presented graphically by plotting the estimated probability of a positive virus result and the pointwise 95% confidence intervals (CI) of the curves. We used repeated 10-fold cross-validation (CV) to estimate the four metrics: Brier score, the *c* concordance index, the calibration intercept and slope.

To evaluate the methods on smaller sample sizes we used 100 subsamples of 500 units. For the splines, at each iteration the number of knots minimizing the training data AIC was used to fit the model, which was evaluated on the remaining units. The proportion of models for which the overall score test had a *p*-value less than 0.05 was also recorded (referred as power in the following). To evaluate the effect of this type of knots selection we reported also the results obtained using a fixed number of knots. The simulation was repeated using a fixed number of knots (equal to the median optimal number of knots obtained in the previous simulation), or varying the number of knots (between 3 (5 for periodic RCS) and 10). hPIV1 and hPIV2 were excluded from these analyses because of their low event rate.

In our analyses with periodic RCS and CS we used the first week of the year as the starting point of the calendar year period, which is a common but arbitrary choice. We evaluated the impact of the choice of the period’s starting point on the estimated probabilities: we re-analyzed the complete data set and its subsets and changed the starting point (setting it to the 1st week, the 4th, 8th, 12th, …, 52nd week), estimating 14 different models in each setting. For the complete data set we analyzed all the viruses, using 7 and 10 knots and graphically displayed the estimated probabilities obtained using the 14 different starting points. In the subset analysis we used randomly selected subsamples of 500 observations, considered 5 to 10 knots and analyzed the RSV, AdV, hMPV and Flu viruses; for each unit included in the subsample we evaluated the standard deviation (SD) of the estimated probabilities obtained with the 14 different models and calculated the average SD. The analyses were repeated 500 times and the results were averaged. In our applications the position of the knots was fixed at specific quantiles, therefore changing the starting point had also the effect of changing the location of the knots.

### Simulation studies

We performed two sets of simulations to evaluate the performance of periodic RCS and CS and to compare them to those obtained with RCS and the cosinor models with one and two harmonics. In the first sets (alternative cases) there was an association between the periodic explanatory variable and the binary outcome, in the second (null case) there was no association; data were analyzed with logistic regression.

The periodic explanatory variable *x* was simulated from a Uniform distribution defined in the [0, 1] interval (*U*(0, 1)). For each subject we evaluated the probability of experiencing the event from a known model (*P*(*Y* = 1|*x*) = (1 + sin(2*πx*)) ⋅ 0.25 + 0.25 in the first alternative case, *P*(*Y* = 1|*x*) = (13 + 3 ⋅ sin(2*πx*) + cos(2*πx*) − 20 ⋅ sin(2*πx*) ⋅ cos(2*πx*))/25 in the second alternative case, *P*(*Y* = 1|*x*) = (2.5 − *sin*(2*πx*) + 1.1 ⋅ cos(*x*) − *sin*(2*πx*) ⋅ cos(*x*))/7 + 0.2 in the third alternative case; *x* and the outcome were independent in the null case, *P*(*Y* = 1|*X*) = 0.5).

We simulated the occurrence of the event if this probability was greater than a random number generated from *U*(0, 1). The probabilities were simulated from a one-harmonic sinusoidal function in the first alternative case, in the second alternative case the probabilities had a bimodal distribution, while the function was bimodal with a flat region in the third alternative case. The shape of the functions used to generate the probabilities are depicted as black curves in Fig 2.

In the first alternative case we used a fixed number of knots (5) or parameters (3) for the splines; in the other two cases we used a fixed number of parameters (3 or 5). For the alternative cases the data used to develop the model included 100 units, test data included 1000 units and we evaluated the four previously described measures. Additionally, we presented the results graphically, showing the average estimated curve and the average 95% pointwise CI, the curves estimated in a subsample of the simulation iterations, and the coverage of the 95% pointwise CI for the linear predictor, LP (the proportion of simulations where the CI included the true simulated value, dividing the range of the explanatory variable in equally spaced intervals of 0.025 width and evaluating the coverage in their mid-point). The average coverage and the average length of the 95% CI for the LP was obtained averaging the values from the intervals. For the alternative and the null cases we evaluated the probability that the *p*-value obtained from the score or likelihood ratio test (LRT) for the overall association between the explanatory variable and the outcome was less than 0.05. This probability estimates the power in the alternative case and the size of the test (probability of false positives) in the null case, using the level of significance *α* = 0.05. All simulations were repeated 5000 times for the alternative case, 20000 to evaluate the size of the tests in the null case. R version 3.3.1 was used and the random seed was 1234 for all the simulations.

## Results

### Reanalysis of the data from Horton et al

**Fig 1:**
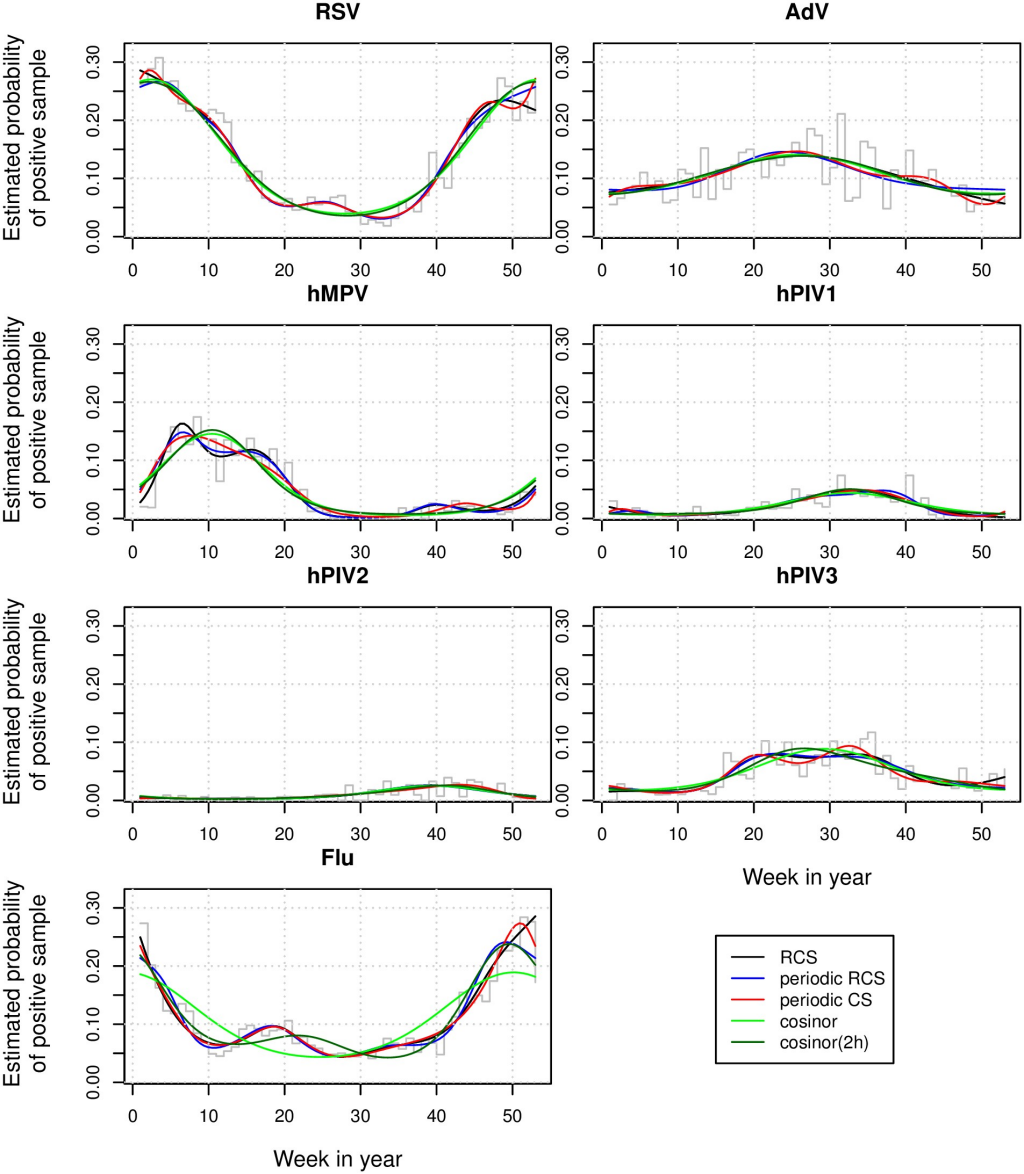
Estimated probability of virus positivity for 7 different viruses using RCS, periodic RCS and CS, and cosinor models with one and two harmonics using data from Horton et al. The step functions are the observed weekly proportions of virus positive samples, the lines are the estimated probabilities estimated using the 5 different models.

The major differences between the estimated probabilities from the five models were observed in the extremes (Fig 1). As expected, the estimated values at the beginning and at the end of the calendar year did not match for RCS, while they did match and the function was smooth with periodic RCS and CS and for the cosinor models; for example, with RCS the estimated probability of RSV positivity was 0.29 on January 1 and 0.22 on December 31, while they were 0.26 with periodic RCS and the cosinor model with 2 harmonics, and 0.27 with periodic CS and with the cosinor model. The 95% CI in the extremes were much wider when RCS were used, the cosinor model had the narrowest CI, followed by periodic RCS (S1 Fig). The optimal number of knots (minimizing AICs) was smaller for the viruses with smaller sample sizes, periodic RCS required the estimation of fewer parameters (but not necessarily fewer knots), except for hPIV1. The seasonal modelling of hMPV achieved the best discrimination, AdV the worse.

The differences in cross-validated Brier score and *c* index between the methods were mostly negligible and the models were well calibrated (Table 1). The spline models captured the bimodality for INF, hMPV and hPIV3, cosinor(2h) only for INF; for these viruses the simple cosinor model performed worse in terms of calibration (Fig 1).

**Table 1:** Analysis of the complete dataset of Horton et al. Estimates are obtained with repeated 10-fold cross-validation.

Virus	Method	Parameters	Knots	Brier score	*c* index	Cal. intercept	Cal. slope
RSV (n = 24503, 16.1% Pos)	RCS	9	10	0.127	0.680	-0.005	0.997
	RCS Per	7	10	0.127	0.680	-0.002	0.999
	CS Per	10	10	0.127	0.679	-0.007	0.996
	cosinor	2		0.127	0.678	0.004	1.004
	cosinor(2h)	4		0.127	0.677	0.004	1.002
AdV (n = 9402, 9.8% Pos)	RCS	4	5	0.088	0.574	-0.068	0.970
	RCS Per	2	5	0.088	0.563	-0.007	0.998
	CS Per	8	8	0.088	0.574	-0.219	0.901
	cosinor	2		0.089	0.574	-0.010	0.997
	cosinor(2h)	4		0.089	0.572	-0.098	0.957
hMPV (n = 9384, 6.6% Pos)	RCS	8	9	0.059	0.739	-0.009	0.999
	RCS Per	6	9	0.059	0.738	0.013	1.009
	CS Per	7	7	0.059	0.737	0.004	1.005
	cosinor	2		0.059	0.726	0.056	1.028
	cosinor(2h)	4		0.059	0.725	0.022	1.012
hPIV1 (n = 9402, 1.7% Pos)	RCS	4	5	0.017	0.707	0.008	1.012
	RCS Per	7	10	0.017	0.696	-0.194	0.957
	CS Per	8	8	0.017	0.695	-0.257	0.940
	cosinor	2		0.017	0.683	0.125	1.043
	cosinor(2h)	4		0.017	0.668	-0.104	0.981
hPIV2 (n = 9402, 0.9% Pos)	RCS	4	5	0.009	0.699	-0.105	0.986
	RCS Per	2	5	0.009	0.709	0.089	1.030
	CS Per	5	5	0.009	0.704	-0.207	0.963
	cosinor	2		0.009	0.714	0.314	1.087
	cosinor(2h)	4		0.009	0.712	0.069	1.031
hPIV3 (n = 9402, 3.9% Pos)	RCS	7	8	0.037	0.675	-0.091	0.974
	RCS Per	5	8	0.037	0.672	-0.058	0.985
	CS Per	10	10	0.037	0.670	-0.175	0.946
	cosinor	2		0.037	0.667	0.008	1.006
	cosinor(2h)	4		0.037	0.670	-0.048	0.988
INF (n = 28438, 11.8% Pos)	RCS	9	10	0.098	0.674	-0.014	0.993
	RCS Per	7	10	0.099	0.672	-0.010	0.995
	CS Per	10	10	0.098	0.676	-0.015	0.992
	cosinor	2		0.099	0.663	0.007	1.004
	cosinor(2h)	4		0.098	0.665	-0.002	1.000

The results between methods differed more consistently using smaller sample sizes (*n* = 500, Table 2); the estimated median optimal number of knots was smaller compared to the results obtained on complete data. The power of three viruses was consistently close to 1 but periodic RCS had larger power for the viruses with moderate power (AdV and hPIV3). None of the methods performed consistently better in terms of the other four metrics but the estimates obtained using cosinor and periodic RCS were less variable (S2 Fig). Overall, the models using fewer parameters tended to perform better on new data, having less overfitting and achieving smaller errors and higher discrimination.

**Table 2:** Repeated analysis of subsets of 500 units; the number of knots is based on AIC; estimates (Brier score, *c* index, calibration intercept and slope) are obtained on the data not included in the model estimation, power is evaluated on training data.

Virus	Method	Parameters	Knots	Brier score	c index	Calibration intercept	Calibration slope	Power
RSV	RCS	4	5	0.1291	0.667	-0.005	0.786	1.00
	RCS Per	4	7	0.1284	0.667	-0.004	0.816	1.00
	CS Per	3	3	0.1285	0.670	-0.007	0.857	1.00
	cosinor	2		0.1277	0.674	0.007	0.993	1.00
	cosinor(2h)	4		0.1282	0.668	-0.008	0.885	1.00
AdV	RCS	2	3	0.0889	0.560	-0.001	0.477	0.38
	RCS Per	2	5	0.0891	0.546	0.004	0.435	0.43
	CS Per	3	3	0.0892	0.543	0.004	0.399	0.32
	cosinor	2		0.0890	0.556	0.003	0.851	0.37
	cosinor(2h)	4		0.0895	0.542	0.003	0.443	0.27
hMPV	RCS	5	6	0.0600	0.715	-0.020	0.664	1.00
	RCS Per	4	7	0.0597	0.721	0.009	0.734	1.00
	CS Per	6	6	0.0600	0.714	-0.085	0.546	0.99
	cosinor	2		0.0597	0.723	0.020	0.927	0.99
	cosinor(2h)	2		0.0599	0.720	0.032	0.774	0.98
hPIV3	RCS	2	4	0.0374	0.652	-0.020	0.550	0.65
	RCS Per	2	5	0.0374	0.661	-0.000	0.666	0.76
	CS Per	4	4	0.0375	0.648	-0.055	0.465	0.64
	cosinor	2		0.0370	0.649	0.043	0.880	0.63
	cosinor(2h)	4		0.0372	0.638	0.043	0.692	0.51
INF	RCS	3	4	0.0998	0.656	-0.007	0.771	0.97
	RCS Per	5	8	0.1001	0.655	-0.005	0.699	0.94
	CS Per	4	4	0.0995	0.654	-0.006	0.767	0.97
	cosinor	2		0.1000	0.655	0.001	0.999	0.91
	cosinor(2h)	4		0.0995	0.657	-0.001	0.859	0.96

The models obtained without optimizing the number of knots (keeping their number equal to the estimated median optimal number from the previous simulations) had considerably less overfitting (S1 Table): the major differences were the smaller power on training data and better calibration on new data, the performance on new data was somehow better also in terms of Brier score and *c* index.

Generally, in terms of all the considered metrics, periodic RCS outperformed RCS and periodic CS with the same number of fixed knots (S1 File). The only exception was observed for the INF virus, which was modelled poorly with few knots (*n*_*k*_ < 7) with periodic RCS, but not with RCS and periodic CS. The comparison of the results obtained keeping fixed the number of estimated parameters did not give the same clear cut results: periodic RCS had better power for the two viruses with moderate power, while there was no clear winner in terms of the performance of the other metrics. The simple cosinor models had by far the best calibration, even when compared to models with the same number of parameters; the cosinor(2h) models had considerably worse calibration and, marginally, the performance of the models was worse also in terms of the other metrics. The only exception was the INF virus: the cosinor(2h) model captured its bimodal distribution and performed better than the cosinor model.

**Fig 2:**
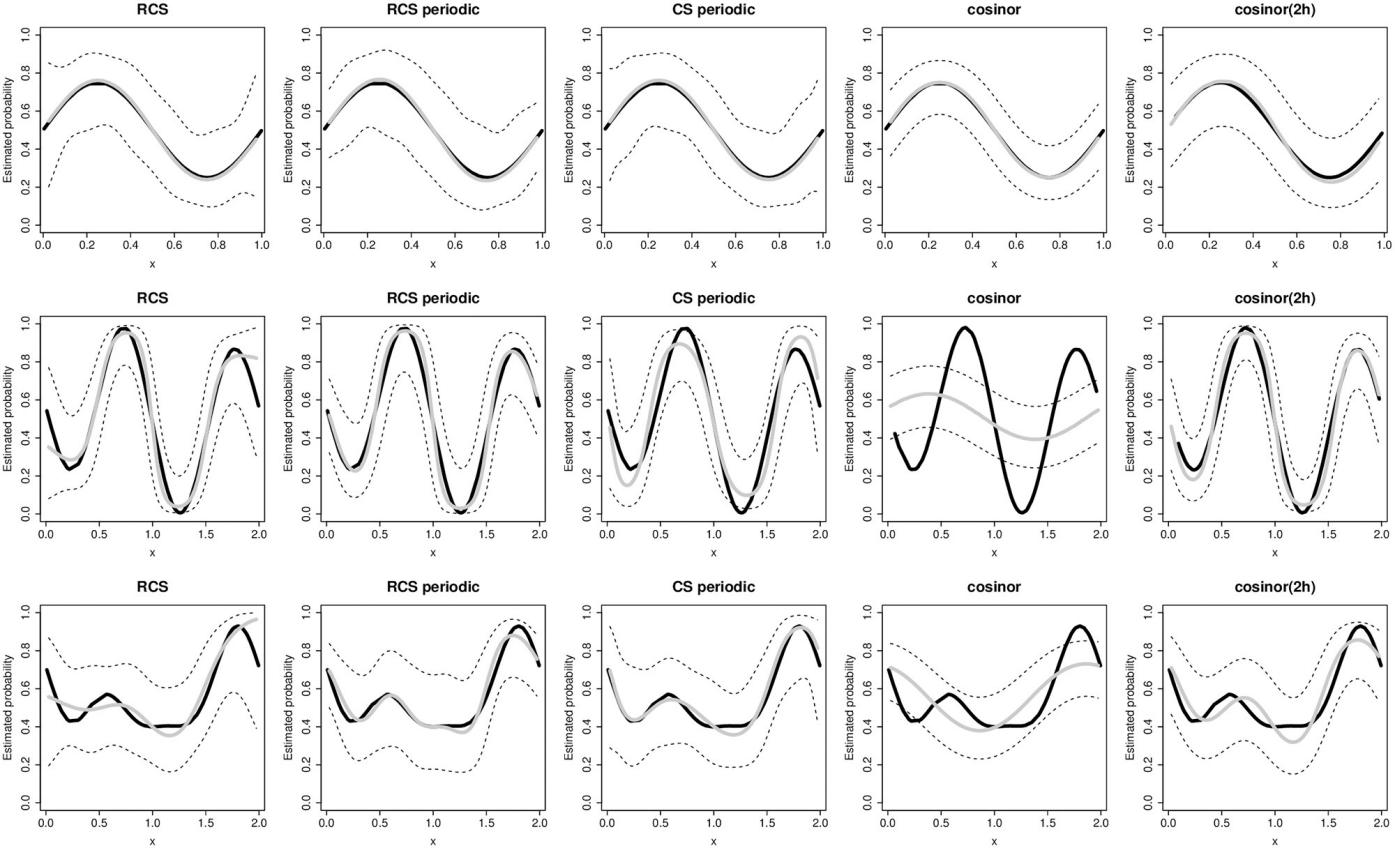
Simulation results for the alternative cases. Each row shows the results from one of the three alternative simulations settings, each column refers to one of the models. The black curves show the probabilities from the model generating the data, the average estimates obtained using the five models are shown with gray lines; dashed lines are average limits of the 95% confidence intervals. Spline models were fitted using 5 parameters. See methods for details on the simulation settings.

As expected, the periodic spline models that used different starting points of the period estimated different probabilities, but the observed differences in our examples were only moderate when we reanalyzed the complete data set (Fig 1 and 2 in S2 File). Overall, the shape of the estimated curves were very similar, regardless of the chosen starting point, and only some of the estimated curves were outlying; the major differences in the estimated probabilities were observed in the starting points of the periods and in their neighborhoods. As expected, the variability among the curves was much bigger when we used a much smaller sample size (examples based on three subsets of 500 units are shown in Fig 3 to 5 in S2 File). The average SD of the estimated probabilities was about 0.01 for AdV, hMPV and INF and about 0.02 for RSV (exact numbers are reported in S2 File). On the complete data set the estimated probabilities obtained with periodic CS had smaller variability compared to those obtained with periodic RCS; the magnitude of the difference between the variability of the two methods depended on the analyzed variable and on the number of used knots (Fig 1 and 2 in S2 File). However, this result does not hold in general, as we observed many situations where periodic RCS had less variability than periodic CS in the paired analyses of the subsamples of 500 units (Table in S2 File).

### Simulation results

The results from the simulations were in line with the findings from real data analysis with small sample sizes. In the first alternative case, when the association between the periodic explanatory variable and the event probability was generated from a one-harmonic sinusoidal function, all three splines types fitted well the simulated data and estimated on average the correct curves, however the estimates obtained with periodic RCS were the least variable (Fig 2 and S3 Fig): their 95% CI were narrower, most notably in the extremes of the period; RCS had the largest CI in the extremes, periodic CS were more variable in the whole range (S3 Fig).

Using a fixed number of 5 knots, periodic RCS performed best on the test data, obtaining slightly smaller Brier score, larger *c* index and perfect calibration, while the estimated calibration slope of RCS and periodic CS indicated overfitting; periodic RCS had the best statistical power, both using LRT and score test, indicating that the true association would be detected more often if periodic RCS were used. When we compared the models using 3 parameters the results were still in favour of periodic RCS, but the differences between models were smaller (Table 3). In this set of simulations the cosinor model had the smallest variability and performed as the best spline model in terms of the other measures (periodic RCS with 5 knots), while the cosinor(2h) model performed considerably worse. This result was in line with the expectations, as the data generating mechanism was consistent with the cosinor model. For all the methods the coverage of the 95% CI was very close to the nominal value over the whole range of the period, the average length of the 95% CI was the shortest for the cosinor model and for periodic RCS with 5 knots (S4 Fig and Table 3).

**Table 3:** Simulation results from the alternative and null case; the probabilities in the alternative case are simulated from a sine function, shown in Fig 2.

	RCS	RCS Periodic	CS Periodic	RCS	RCS Periodic	CS Periodic	cosinor	cosinor(2h)
Knots	5	5	5	4	6	3		
Parameters	4	2	5	3	3	3	2	4
Alternative case								
	RCS	RCS Periodic	CS Periodic	RCS	RCS Periodic	CS Periodic	cosinor	cosinor(2h)
Power (LRT *α* = 0.05)	0.847	0.912	0.814	0.873	0.878	0.874	0.913	0.880
Power (Score *α* = 0.05)	0.836	0.909	0.791	0.867	0.872	0.865	0.910	0.880
Brier Train	0.208	0.213	0.206	0.210	0.210	0.210	0.212	0.204
Brier Test	0.231	0.226	0.233	0.228	0.227	0.228	0.226	0.235
AUC Train	0.721	0.709	0.728	0.716	0.717	0.713	0.710	0.734
AUC test	0.678	0.693	0.673	0.687	0.690	0.684	0.694	0.671
Calibration Intercept	0.001	-0.001	0.002	-0.006	-0.004	0.001	-0.001	0.027
Calibration Slope	0.822	0.998	0.754	0.909	0.920	0.901	0.998	0.734
Coverage of 95% CI	0.957	0.951	0.959	0.951	0.955	0.952	0.955	0.956
Length of 95% CI	0.956	0.749	1.082	0.839	0.872	0.864	0.749	0.996
Null case—size of the test								
LRT, *α* = 0.05	0.059	0.053	0.062	0.056	0.057	0.061	0.055	0.056
Score Test, *α* = 0.05	0.049	0.051	0.048	0.049	0.051	0.049	0.052	0.049

In the second set of the alternative simulations, where the probabilities were generated from a bimodal distribution, the cosinor(2h) model outperformed the cosinor model, which had by far the worse performance among the evaluated models (Table 4). The spline models performed better with 5 parameters, RCS having the best performance among the spline models, with similar results to the cosinor(2h) models. The coverage of the 95% CI was well below the nominal value for all the models, but only slightly for periodic RCS with 5 parameters and cosinor(2h) (S4 Fig and Table 4).

**Table 4:** Simulation results from the second alternative case, where the probabilities are simulated from a periodic function with two peaks, shown in Fig 2.

	RCS	RCS Periodic	CS Periodic	RCS	RCS Periodic	CS Periodic	cosinor	cosinor(2h)
Knots	4	6	3	6	8	5		
Parameters	3	3	3	5	5	5	2	4
Power (LRT *α* = 0.05)	0.976	0.997	0.311	1.000	1.000	1.000	0.337	1.000
Power (Score *α* = 0.05)	0.972	0.997	0.301	1.000	1.000	0.999	0.329	1.000
Brier Train	0.187	0.167	0.231	0.154	0.152	0.157	0.234	0.152
Brier Test	0.203	0.183	0.250	0.179	0.175	0.181	0.248	0.175
AUC Train	0.783	0.826	0.642	0.849	0.854	0.843	0.620	0.855
AUC test	0.764	0.806	0.583	0.815	0.826	0.808	0.583	0.829
Calibration Intercept	0.007	0.007	0.048	0.015	0.017	0.012	0.024	0.005
Calibration Slope	0.943	0.958	0.563	0.855	0.862	0.857	0.814	0.879
Coverage of 95% CI	0.490	0.741	0.806	0.872	0.924	0.806	0.260	0.903
Length of 95% CI	0.954	1.128	1.319	1.409	1.523	1.319	0.709	1.241

Also in the third set of the alternative simulations the spline models with 5 parameters outperformed those with 3. On average RCS and cosinor did not estimate the correct association shape, the other models performed similarly: on average the shape was best captured by periodic RCS, but periodic CS had the best power and cosinor(2h) had the best discrimination and the smallest variability (Table 5). The coverage of the 95% CI was slighly below the nominal value for the spline models with 5 parameters and for the cosinor(2h) model, which had the shortest CI length, followed by perdiodic RCS (S4 Fig and Table 5).

**Table 5:** Simulation results from the third alternative, where the probabilities are simulated from a periodic function with complex pattern, shown in Fig 2.

	RCS	RCS Periodic	CS Periodic	RCS	RCS Periodic	CS Periodic	cosinor	cosinor(2h)
Knots	4	6	3	6	8	5		
Parameters	3	3	3	5	5	5	2	4
Power (LRT *α* = 0.05)	0.787	0.677	0.588	0.749	0.752	0.805	0.595	0.773
Power (Score *α* = 0.05)	0.747	0.661	0.567	0.691	0.721	0.759	0.586	0.748
Brier Train	0.214	0.218	0.222	0.208	0.208	0.206	0.225	0.211
Brier Test	0.232	0.237	0.240	0.236	0.236	0.234	0.239	0.234
AUC Train	0.687	0.682	0.666	0.708	0.715	0.714	0.656	0.703
AUC test	0.643	0.640	0.624	0.640	0.649	0.649	0.630	0.651
Calibration Intercept	0.024	0.040	0.045	0.053	0.052	0.045	0.013	0.040
Calibration Slope	0.860	0.833	0.806	0.676	0.719	0.720	0.940	0.778
Coverage of 95% CI	0.895	0.801	0.765	0.924	0.924	0.951	0.711	0.913
Length of 95% CI	0.909	0.851	0.847	1.242	1.091	1.145	0.726	0.994

In the null case the score test performed better than the LRT, its estimated size being closer to the nominal 0.05 value; LRT rejected the true null hypothesis slightly more often than expected for all the considered methods (Tables 3 and 4).

## Discussion

Restricted cubic splines are commonly used to model non-linear associations in regression models. In principle they can be used also to model the association between numerical periodic explanatory variables and outcomes; however, they do not make any use of the additional information contained in the periodicity. The estimates obtained at the beginning and at the end of the period are not constrained to be equal or similar, nor is the variability of the estimates. In practice, even when the sample size is large, the estimates in the extremes can differ substantially and the estimated function often is neither continuous nor smooth.

In this paper we showed how to use restricted cubic splines with numerical constraints to account for the periodicity of the association between a numerical explanatory variable and an outcome, deriving the basis functions for periodic RCS. Our results constrain the curves and their variances to be periodic functions. The method was implemented in the peRiodiCS R package [12], which includes also the implementation of periodic cubic splines originally proposed by Zhang and colleagues [6]. To the best of our knowledge, few methods are currently available to users of statistical software for flexibly modelling periodic variables in regression models [10]. Periodic CS are implemented in mgcv R package [19], using INLA the cyclicity can be modelled with the RW2 model, which corresponds to a cubic smoothing spline [20], periodic B-splines are implemented in the pbs R package [21].

The peRiodiCS package includes an example data set from virology research [2], which we partly reanalyzed in this paper. One of the aims of the original paper was to describe the seasonality of the pathogens; the authors aggregated the results monthly and displayed the proportion of positives among samples tested for each virus using a barplot with juxtaposed bars, and tested the presence of seasonality in virus positivity using chi-squared tests, analyzing the monthly data. We used periodic RCS to give an alternative graphical representation, estimating the seasonality of the viruses using logistic regression models. The graphical presentation using periodic RCS is more effective, as it uses smooth curves and the confidence intervals provide additional important information. An additional advantage is that the statistical inference is statistically more powerful, as it uses fewer degrees of freedom and avoids the categorization of the week of diagnosis into months.

We analyzed real data and performed some simulations to evaluate the extent of the practical impact of the additional constraints on the estimated models. We observed that periodic RCS, besides obtaining continuous and smooth estimates in the extremes of the period, considerably reduces the variability of the estimates for the values close to extremes. With RCS these estimates are known to have the largest variability, therefore using the information about periodicity has an important effect. The estimates obtained with periodic CS are more variable, both at the extremes and, to a lesser extent, in the whole range of values.

To compare the models we additionally used four widely used measures that quantify the overall discrimination and calibration of the models. These measures are less likely to differ substantially between the three types of models, as we observed that the estimates obtained away from the extremes are generally very similar, especially with large samples. However, we observed some systematic differences. When the number of knots is fixed, in most cases periodic RCS outperform RCS and periodic CS, obtaining smaller errors on new data, better discrimination, considerably less overfitting and larger power. From the theoretical point of view these findings can be explained by the use of a smaller number of parameters and by the smaller variability of periodic RCS in the extremes. Comparing models that use the same number of parameters, generally periodic RCS perform better in terms of power, while none of the models outperforms the others based on the other metrics. In the simulations presented in the paper, periodic RCS perform slightly better than the other models also when the number of parameters is fixed, however different simulation settings might favour the other models.

Based on our results with logistic regression, we recommend the use of the score test over the likelihood ratio test to test the overall association between the spline-transformed variables and the outcome, as the likelihood ratio test is slightly anti-conservative, possibly producing more false positive associations than expected.

A critical issue in the use of splines is the choice of the number of knots, which can strongly impact the modelling results. Some of our examples estimated the optimal number of knots minimizing AIC, as suggested by Harrell [1]. Overall, for periodic RCS generally we estimated that the optimal number of parameters was equal or smaller compared to RCS or periodic CS, with the exception of the INF virus, where we found that periodic RCS performed much worse than the other models with few knots, and similarly afterwards. It is therefore possible that while it is suggested that four or five knots are usually adequate when using RCS [9], periodic RCS might require a few more knots in some specific cases.

While the AIC based estimation of the number of knots might be useful from the practical point of view, the results have to be interpreted with care. Our examples show that the optimization of the number of knots introduces a substantial overfitting in the estimated models, which should be accounted for. For example, cross-validation that includes the optimization of the number of knots can be used to obtain nearly unbiased estimates of the performance of the models.

A possible concern in using periodic RCS and CS is the need to choose the starting point of the period, which in many applications can be arbitrary, and the choice of which influences the estimates. This issue might seem even more concerning for periodic RCS that use linear functions beyond boundary knots, where the starting point is located. Nevertheless, the real data examples that we explored indicate a small variability of the estimated probabilities for big samples, which increases for smaller samples and depends on the number of used knots and on the overfitting of the models. The robustness of the choice of the starting point could be assessed by trying different starting points, avoiding to use those that produce outlying estimates.

The greatest variability is present in the neighbourhood of the starting point; therefore, one should avoid starting points that are of specific interest, if the choice is arbitrary. Similarly, if previous knowledge suggests that it is not sensible to assume linearity in a specific time range, starting points included in such intervals should be avoided; this might be the case when changes in the outcome are expected at specific time values. We suggest to place very few observations beyond the boundary knots, as in RCS, thus modelling linearly only a very small part of the data.

We compared the results obtained with the different types of splines with those from the cosinor model, which uses a sine and cosine transformation of the time explanatory variable to model periodic outcomes. The simple cosinor model with two parameters is very parsimonious: it guarantees the equality and smoothness of the estimates at the extremes of the period, and it does not require specialized software; from the theoretical point of view, its most important advantage over periodic RCS and CS is that the choice of the starting point does not have any impact on the results. Extensions of the cosinor models that use more parameters must be used to capture associations that have more than one local minimum and/or maximum. The simple cosinor model outperforms the others in terms of calibration and generally performs well also in terms of the other metrics and has less variability but, as expected, it does not capture well complex association patterns that on the other hand, can be modelled well with splines with few parameters. At the same time, using more harmonics when not needed can considerably worsen the performance of the model. Therefore, also with the cosinor model some care is needed in the choice of the model complexity, raising issues which are similar to those related to the choice of the number of knots for splines.

Our proposal has some limitations, mainly related to its general applicability for periodic data. Periodic RCS, as periodic CS and cosinor models, should not be used if the assumption of equality and smoothness of the outcome at the beginning and at the end of the period is not sensible. For example, discontinuities at the end of the menstrual cycle are scientifically reasonable for basal temperature, which drops considerably at the end of the cycle; none of the periodic models considered in this paper can capture this behaviour, while RCS or CS could be useful for this type of data. Another situation where the continuity and smooth assumption might fail is in the presence of a strong increasing or decreasing trend over time. For example, the deaths attributed to pneumonia and influenza in Brasil decreased on average 2.1 percent annually between 1979 and 2001 [5] and the authors detrended the time series to remove year-to-year variations while preserving seasonal variations, before using Fourier decomposition to describe the amplitude and timing of annual and semiannual epidemic cycles. Similarly, also when periodic RCS is used, it is advisable that existing non-negligible trends are estimated and removed from the data. Examples of detrending methods and their usage can be found in [22].

## Conclusion

We believe that periodic RCS provide a useful expansion of RCS for periodic data, which can enhance data visualization and be a useful tool for regression modelling of periodic variables.

## Supporting information

S1 Fig Estimated probability of RSV positivity using the complete dataset. The methods reported in the Figure are RCS (black line), periodic RCS (blue), periodic CS (red), cosinor (light green) and cosinor(2h) (dark green) with 95% pointwise confidence intervals (dashed lines). Estimates are obtained using the complete dataset.
(PDF)

S2 Fig Estimated probability of RSV positivity using subsets. The methods reported in the Figure are RCS, periodic RCS and periodic CS, cosinor and cosinor(2h). Estimates are obtained using randomly drawn subsets of 500 units from the complete dataset.
(PDF)

S3 Fig Variability of the simulation results in the alternative setting. True (black) and estimated (dashed) curves for a random subsample of the simulations in the three alternative setting. Spline models were fitted using 5 parameters.
(PDF)

S4 Fig Coverage of the 95% CI from the simulations in the alternative settings. Simulation results using 5 parameters for the spline models. Coverage of the 95% CI in sub-intervals for models using splines. Rows are the three alternative simulation settings, columns the five different models. Note the different scale in the first row.
(TIFF)

S1 Table Results from the repeated analysis of 500 units using the *optimal number of knots*. The number of knots is kept fixed at the median number of optimal knots estimated in the analysis presented in Table 2. Estimates (Brier score, *c* index, calibration intercept and slope) are obtained on the data not included in the model estimation process; power is evaluated on traning data.
(PDF)

S1 File Results from the repeated analysis of 500 units varying the number of knots or parameters. Power is estimated on training data, the other metrics on new data. The results are presented as a function of the number of knots used or the number of estimated parameters.
(PDF)

S2 File Effect of the choice of the starting point of the period for periodic RCS and CS. Estimated probabilities obtained from analyses that use different starting points for the periods; the analyses are based on the complete data set of Horton et al. and on the repeated analysis of 500 units.
(PDF)

S1 Appendix (PDF)
