# Supplementary figures and images for: Restricted cubic splines for modelling periodic data
Source: PLoS One. 2020 Oct 28;15(10):e0241364. doi: 10.1371/journal.pone.0241364 (PMC7592770; doi:10.1371/journal.pone.0241364)

# RSV

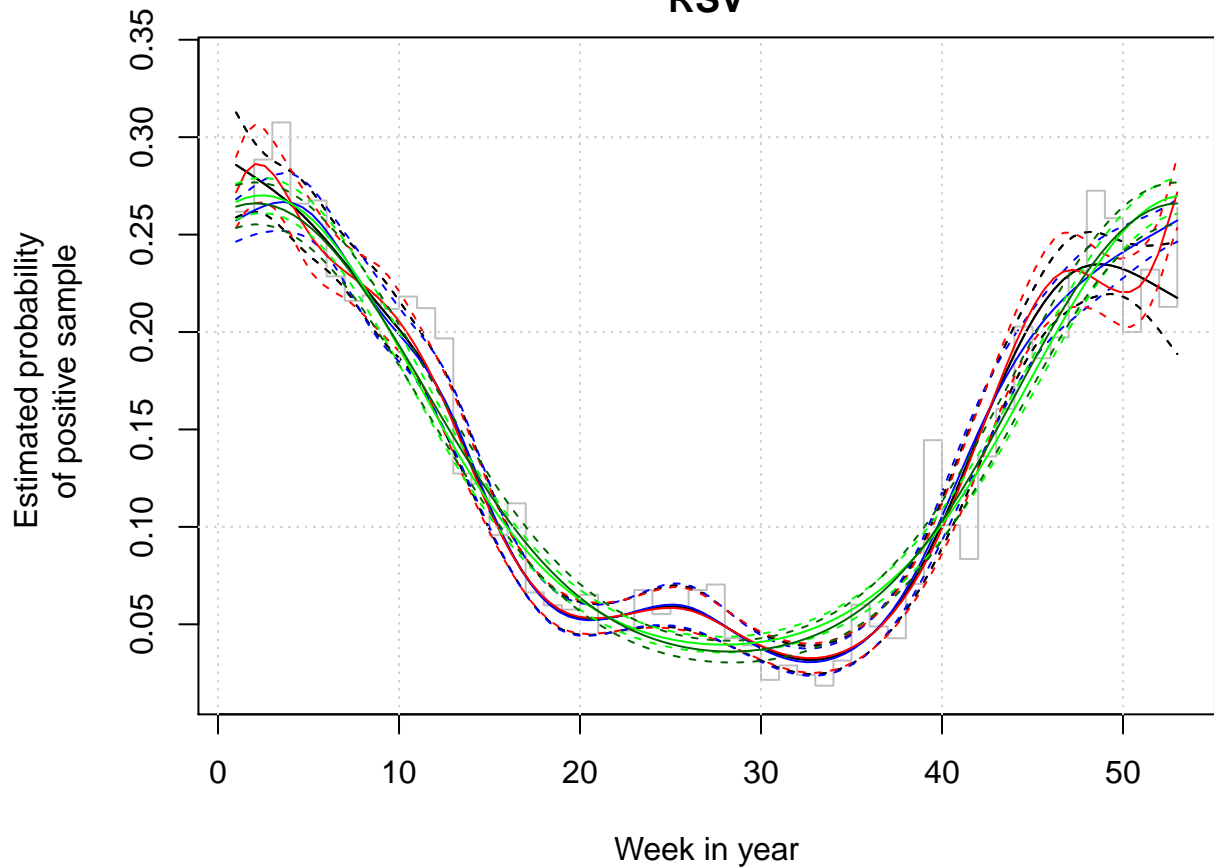

Supplement: S1 Fig — The methods reported in the Figure are RCS (black line), periodic RCS (blue), periodic CS (red), cosinor (light green) and cosinor(2h) (dark green) with 95% pointwise confidence intervals (dashed lines). Estimates are obtained using the complete dataset. (PDF) [file pone.0241364.s001.pdf]

**RSV – RCS**

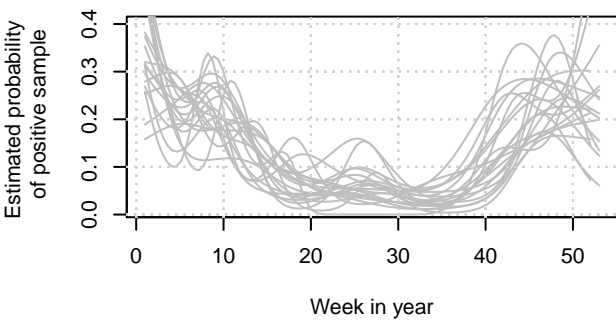

**RSV – RCS.PER**

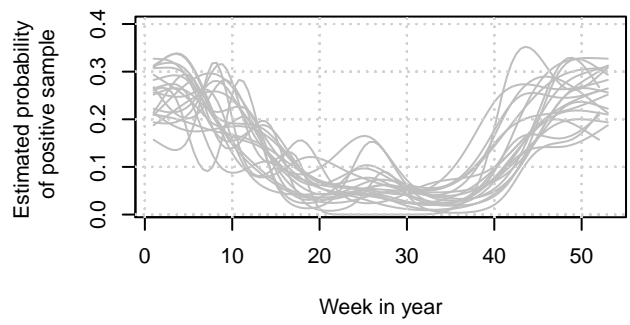

**RSV – CS.PER**

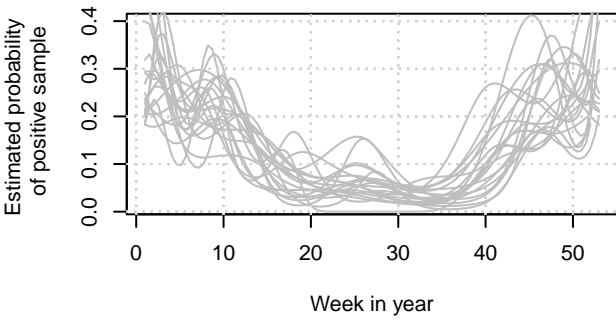

**RSV – cosinor**

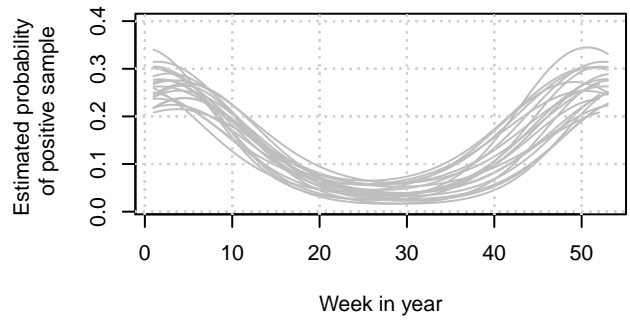

**RSV – cosinor(2h)**

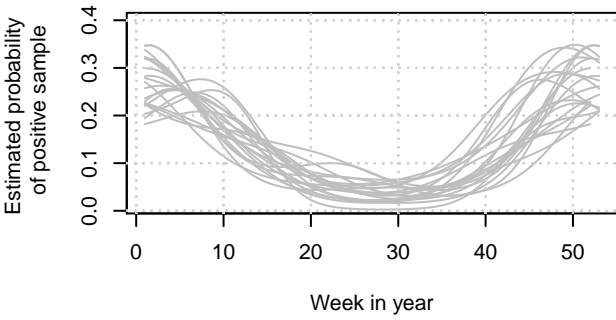

Supplement: S2 Fig — The methods reported in the Figure are RCS, periodic RCS and periodic CS, cosinor and cosinor(2h). Estimates are obtained using randomly drawn subsets of 500 units from the complete dataset. (PDF) [file pone.0241364.s002.PDF]

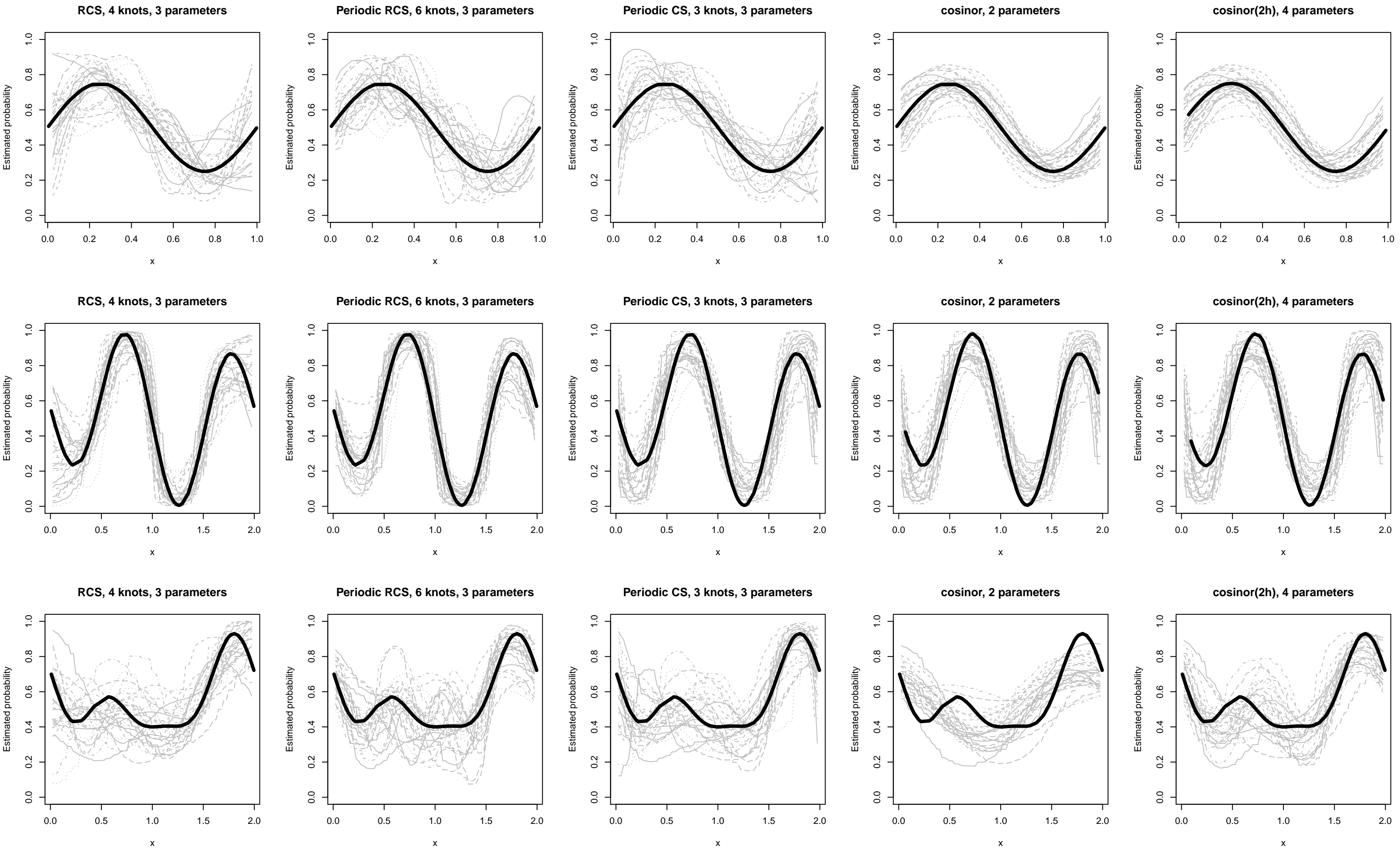

Supplement: S3 Fig — True (black) and estimated (dashed) curves for a random subsample of the simulations in the three alternative setting. Spline models were fitted using 5 parameters. (PDF) [file pone.0241364.s003.PDF]

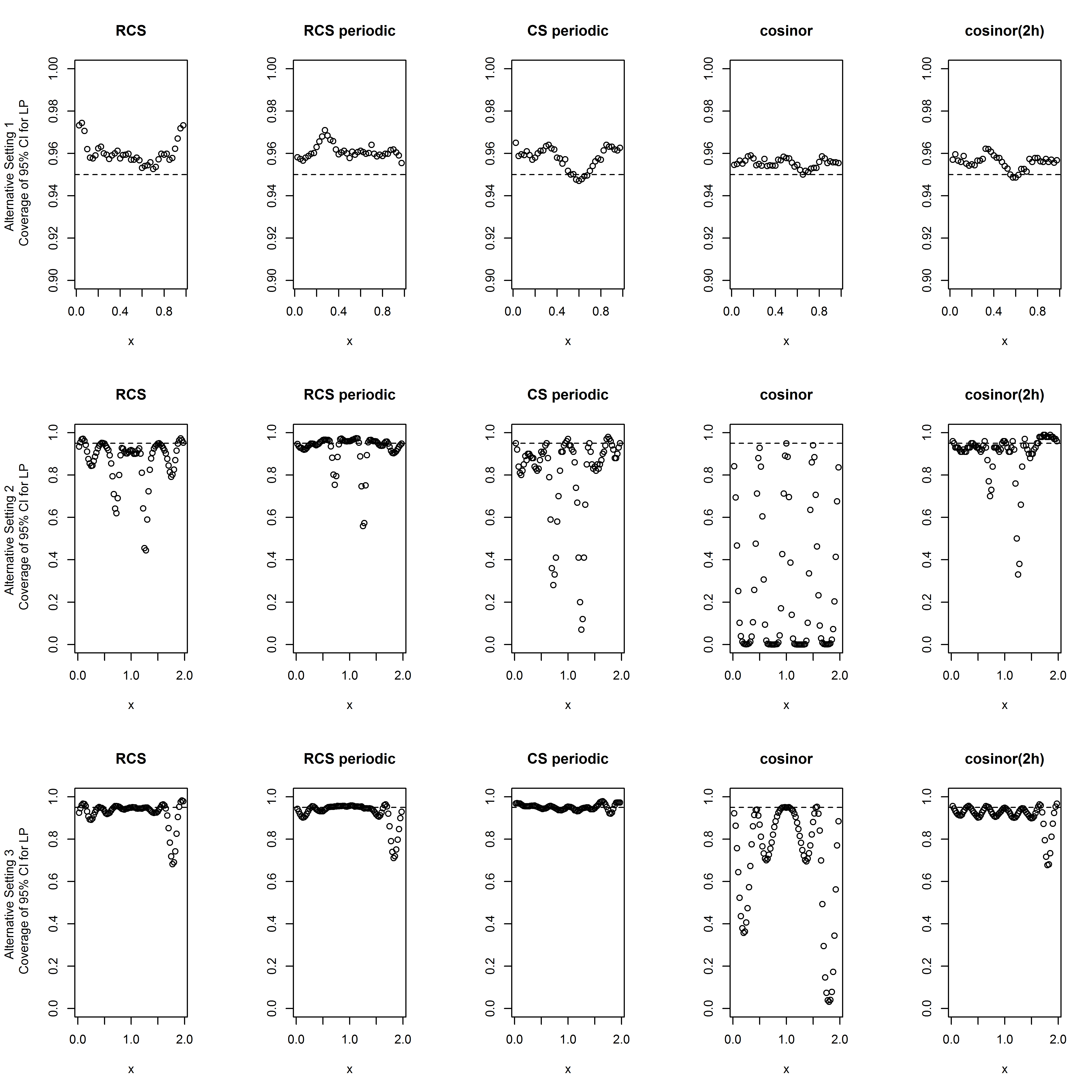

Supplement: S4 Fig — Simulation results using 5 parameters for the spline models. Coverage of the 95% CI in sub-intervals for models using splines. Rows are the three alternative simulation settings, columns the five different models. Note the different scale in the first row. (TIFF) [file pone.0241364.s004.tiff]
