# Supplementary material for: Restricted cubic splines for modelling periodic data
Source: PLoS One. 2020 Oct 28;15(10):e0241364. doi: 10.1371/journal.pone.0241364 (PMC7592770; doi:10.1371/journal.pone.0241364)
Supplement: S1 Table — The number of knots is kept fixed at the median number of optimal knots estimated in the analysis presented in Table 2. Estimates (Brier score, c index, calibration intercept and slope) are obtained on the data not included in the model estimation process; power is evaluated on traning data. (PDF) [file pone.0241364.s005.pdf]

Table 1: Additional File 3. Repeated analysis of subsets of 500 units; the number of knots fixed at the value obtained minimizing AIC; estimates are obtained on the data not included in the model estimation.

Virus	Method	Parameters	Knots	Brier score	c index	Calibration intercept	Calibration slope	Power
RSV	RCS	4	5	0.1286	0.670	-0.007	0.908	1.00
	RCS Per	4	7	0.1281	0.670	-0.004	0.894	1.00
	CS Per	3	3	0.1281	0.673	-0.007	0.937	1.00
	cosinor	2		0.1277	0.674	0.007	0.993	1.00
	cosinor(2h)	4		0.1282	0.668	-0.008	0.885	1.00
AdV	RCS	2	3	0.0887	0.566	0.001	0.688	0.27
	RCS Per	2	5	0.0887	0.555	0.006	0.601	0.31
	CS Per	3	3	0.0889	0.552	0.004	0.508	0.25
	cosinor	2		0.0890	0.556	0.003	0.851	0.37
	cosinor(2h)	4		0.0895	0.542	0.003	0.443	0.27
hMPV	RCS	5	6	0.0599	0.715	0.041	0.762	0.97
	RCS Per	4	7	0.0596	0.723	0.052	0.803	0.98
	CS Per	6	6	0.0597	0.718	0.027	0.721	0.99
	cosinor	2		0.0597	0.723	0.020	0.927	0.99
	cosinor(2h)	4		0.0372	0.638	0.043	0.692	0.51
hPIV3	RCS	2	4	0.0372	0.654	0.025	0.621	0.47
	RCS Per	2	5	0.0372	0.668	0.025	0.800	0.74
	CS Per	4	4	0.0373	0.655	0.016	0.618	0.53
	cosinor	2		0.0370	0.649	0.043	0.880	0.63
	cosinor(2h)	4		0.0372	0.638	0.043	0.692	0.51
INF	RCS	3	4	0.0995	0.659	-0.009	0.845	0.95
	RCS Per	5	8	0.0999	0.658	-0.008	0.750	0.92
	CS Per	4	4	0.0993	0.656	-0.002	0.809	0.95
	cosinor	2		0.1000	0.655	0.001	0.999	0.91
	cosinor(2h)	4		0.0995	0.657	-0.001	0.859	0.96
