# Supplementary material for: Restricted cubic splines for modelling periodic data
Source: PLoS One. 2020 Oct 28;15(10):e0241364. doi: 10.1371/journal.pone.0241364 (PMC7592770; doi:10.1371/journal.pone.0241364)
Supplement: S1 File — Power is estimated on training data, the other metrics on new data. The results are presented as a function of the number of knots used or the number of estimated parameters. (PDF) [file pone.0241364.s006.pdf]

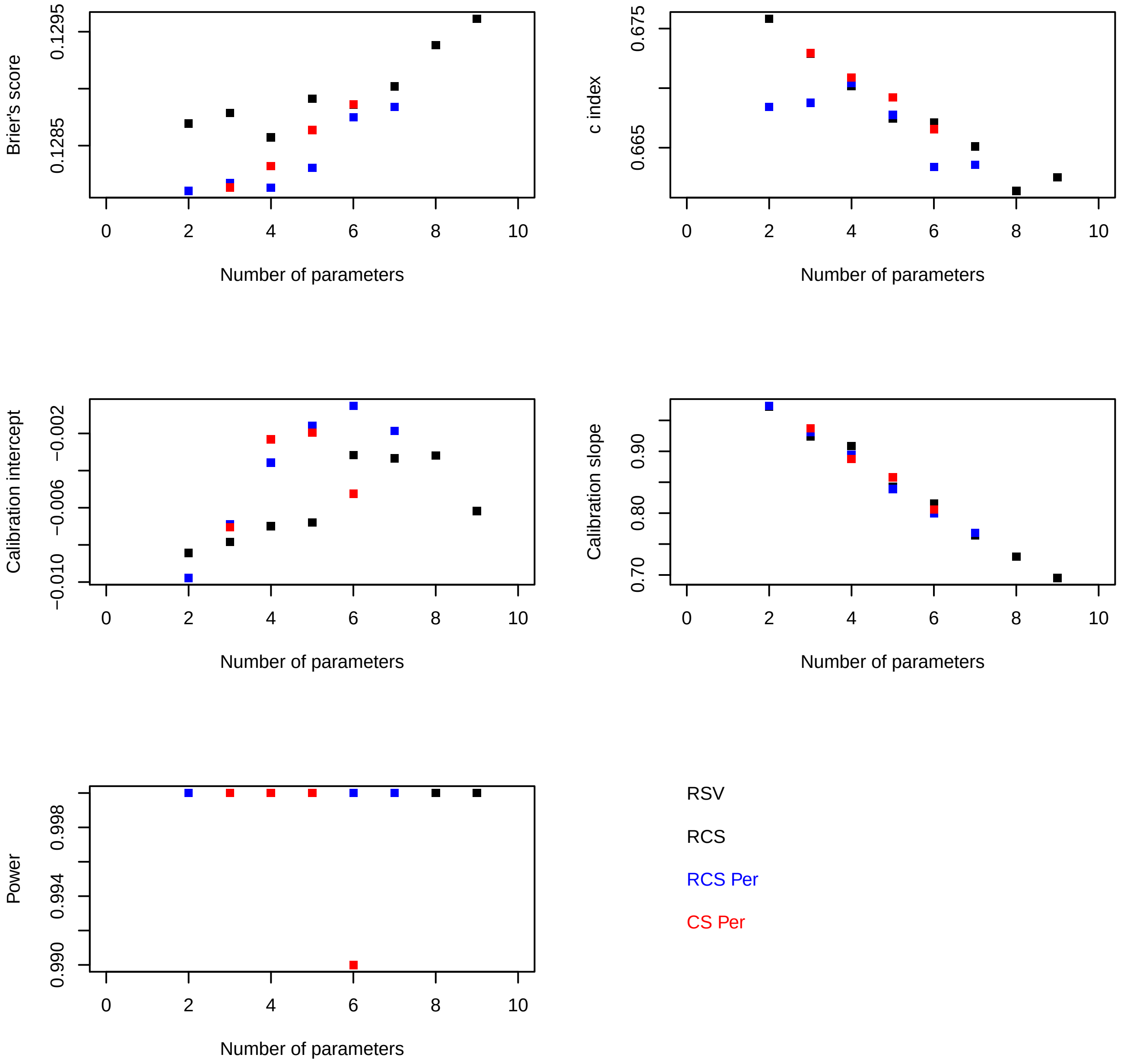
Brier's score
0.1285
0.1295
c index
0.665
0.675
Calibration intercept
−0.010
−0.006
−0.002
Calibration slope
0.70
0.80
0.90
Power
0.990
0.994
0.998
0
2
4
6
8
10
Number of parameters
RSV
RCS
RCS Per
CS Per

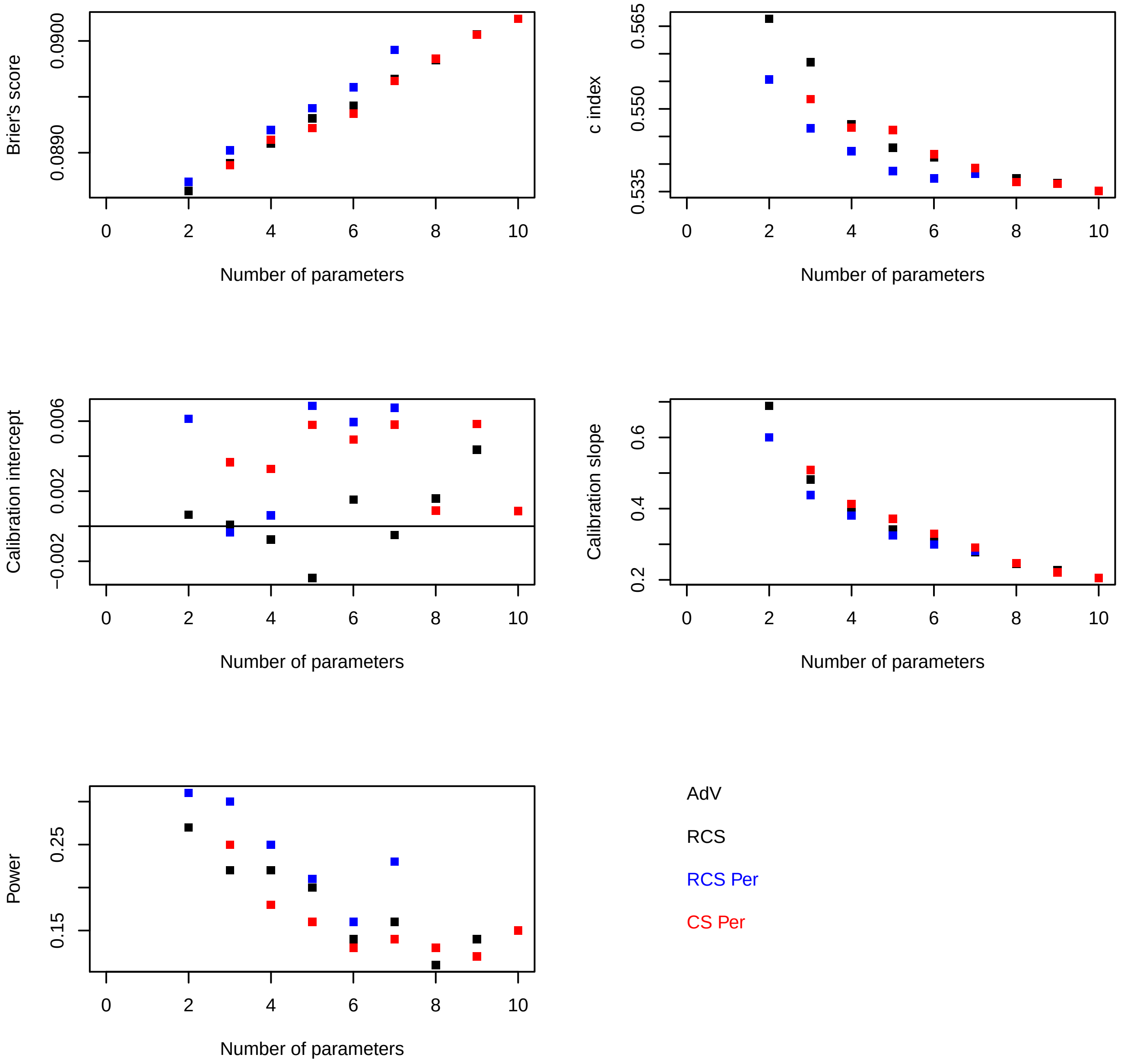
Brier's score
0.0890
0.0900
c index
0.535
0.550
0.565
Calibration intercept
−0.002
0.002
0.006
Calibration slope
0.2
0.4
0.6
Power
0.15
0.25
0
2
4
6
8
10
Number of parameters
AdV
RCS
RCS Per
CS Per

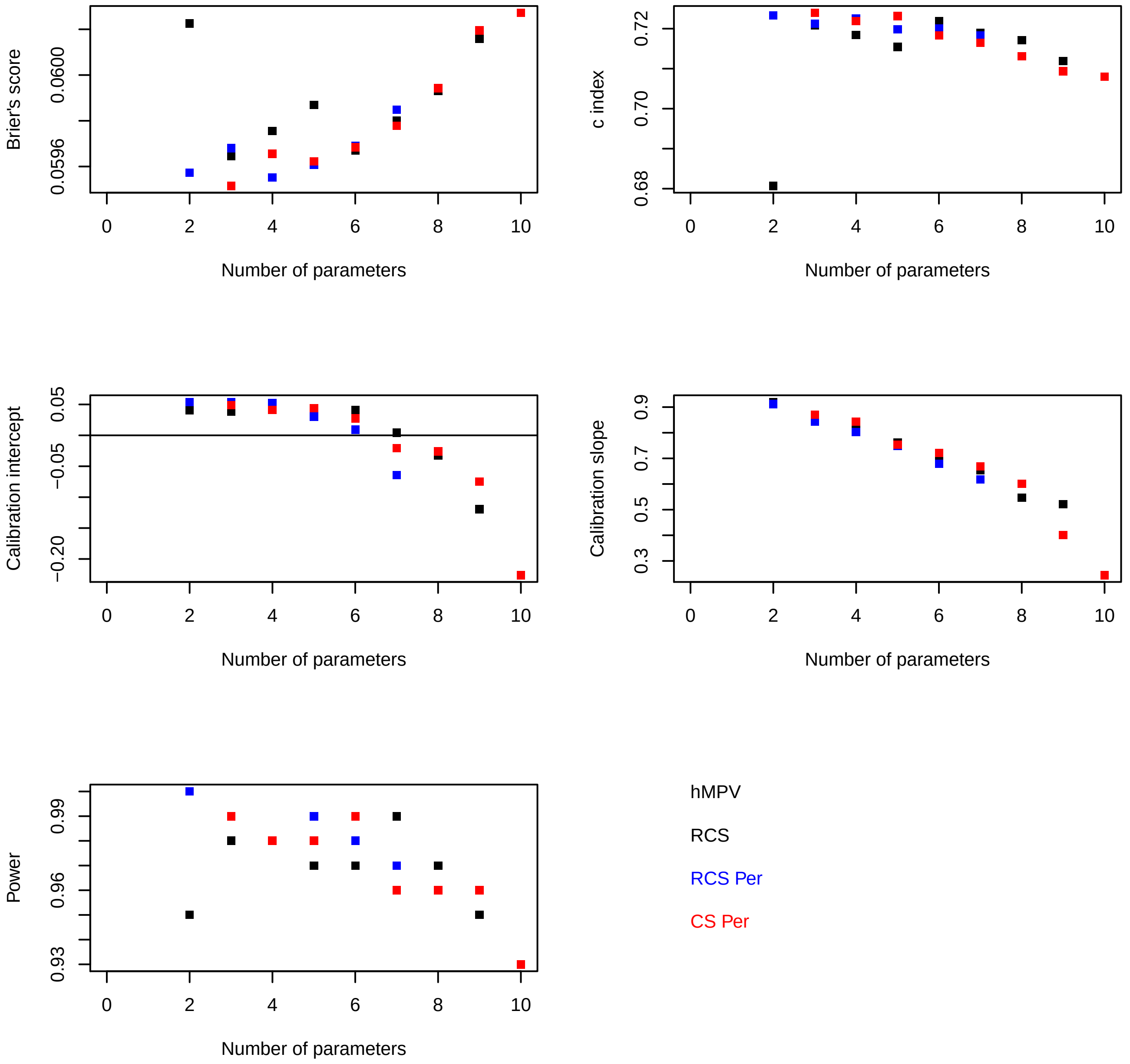

Brier's score
0.0596
0.0600
c index
0.68
0.70
0.72
Calibration intercept
−0.20
−0.05
0.05
Calibration slope
0.3
0.5
0.7
0.9
Power
0.93
0.96
0.99
0
2
4
6
8
10
Number of parameters
hMPV
RCS
RCS Per
CS Per

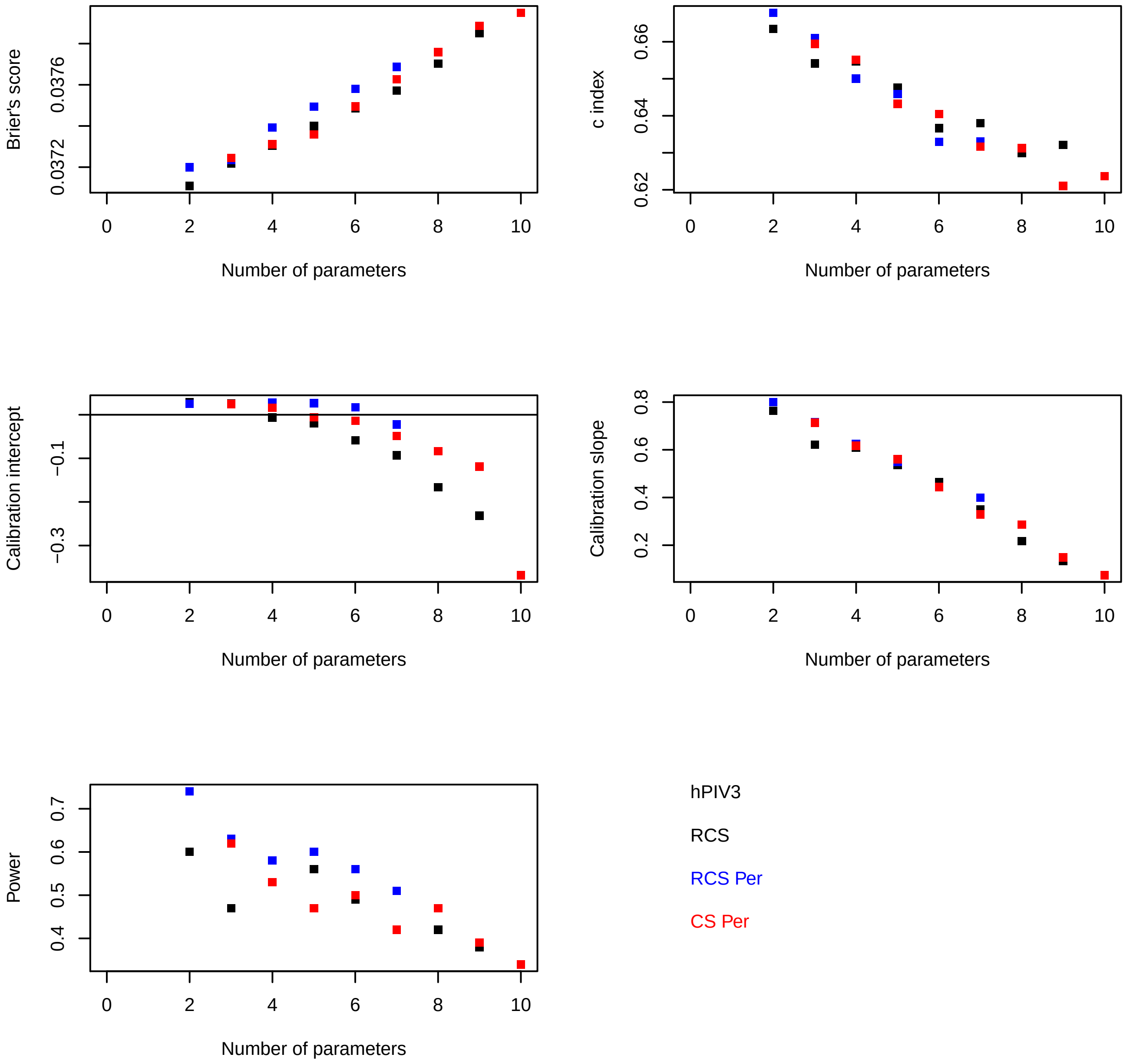

Brier's score
0.0372
0.0376
c index
0.62
0.64
0.66
Calibration intercept
−0.3
−0.1
Calibration slope
0.2
0.4
0.6
0.8
Power
0.4
0.5
0.6
0.7
0
2
4
6
8
10
Number of parameters
hPIV3
RCS
RCS Per
CS Per

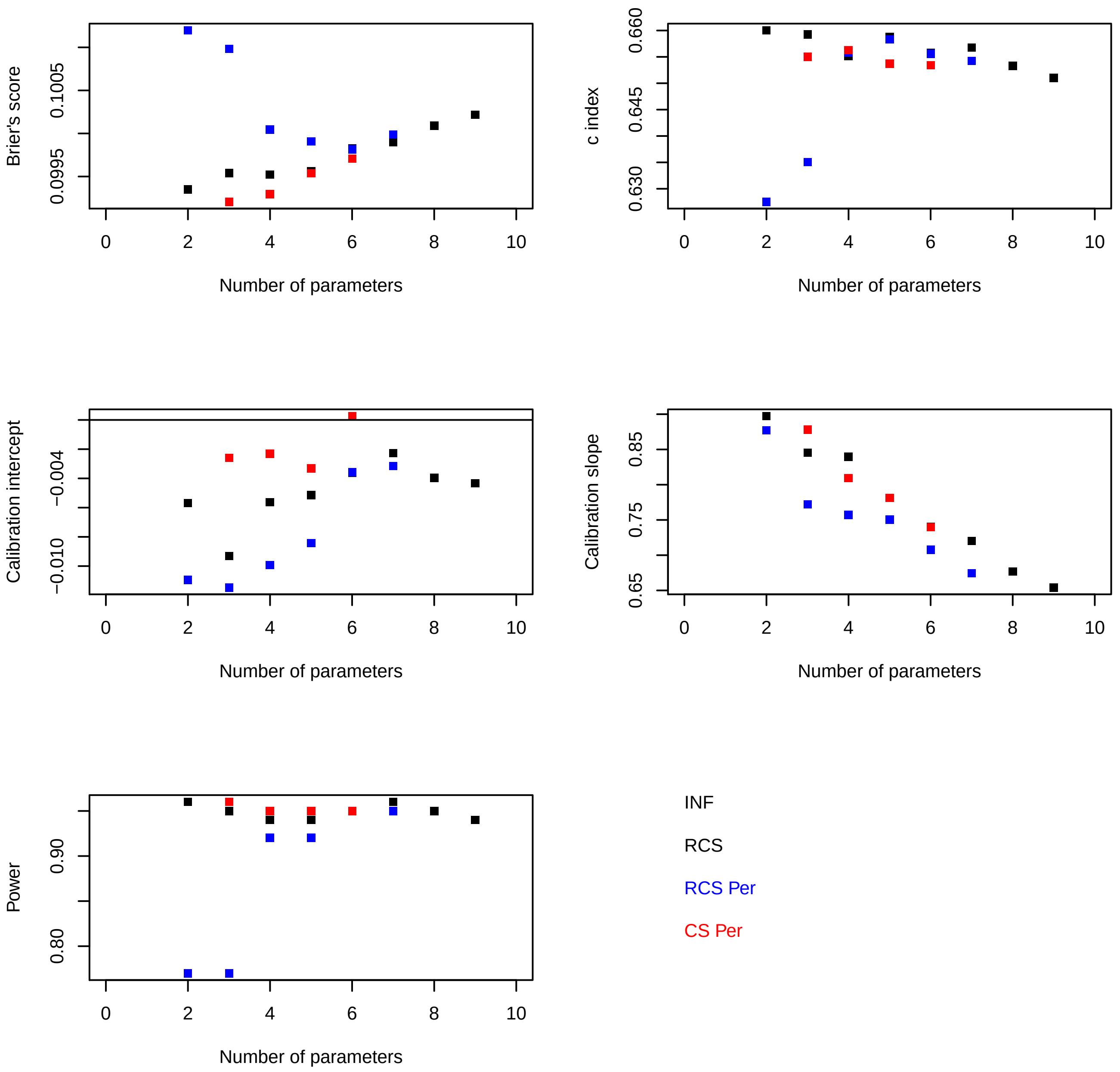
Brier's score
0.0995
0.1005
c index
0.630
0.645
0.660
Calibration intercept
−0.010
−0.004
Calibration slope
0.65
0.75
0.85
Power
0.80
0.90
0
2
4
6
8
10
Number of parameters
INF
RCS
RCS Per
CS Per

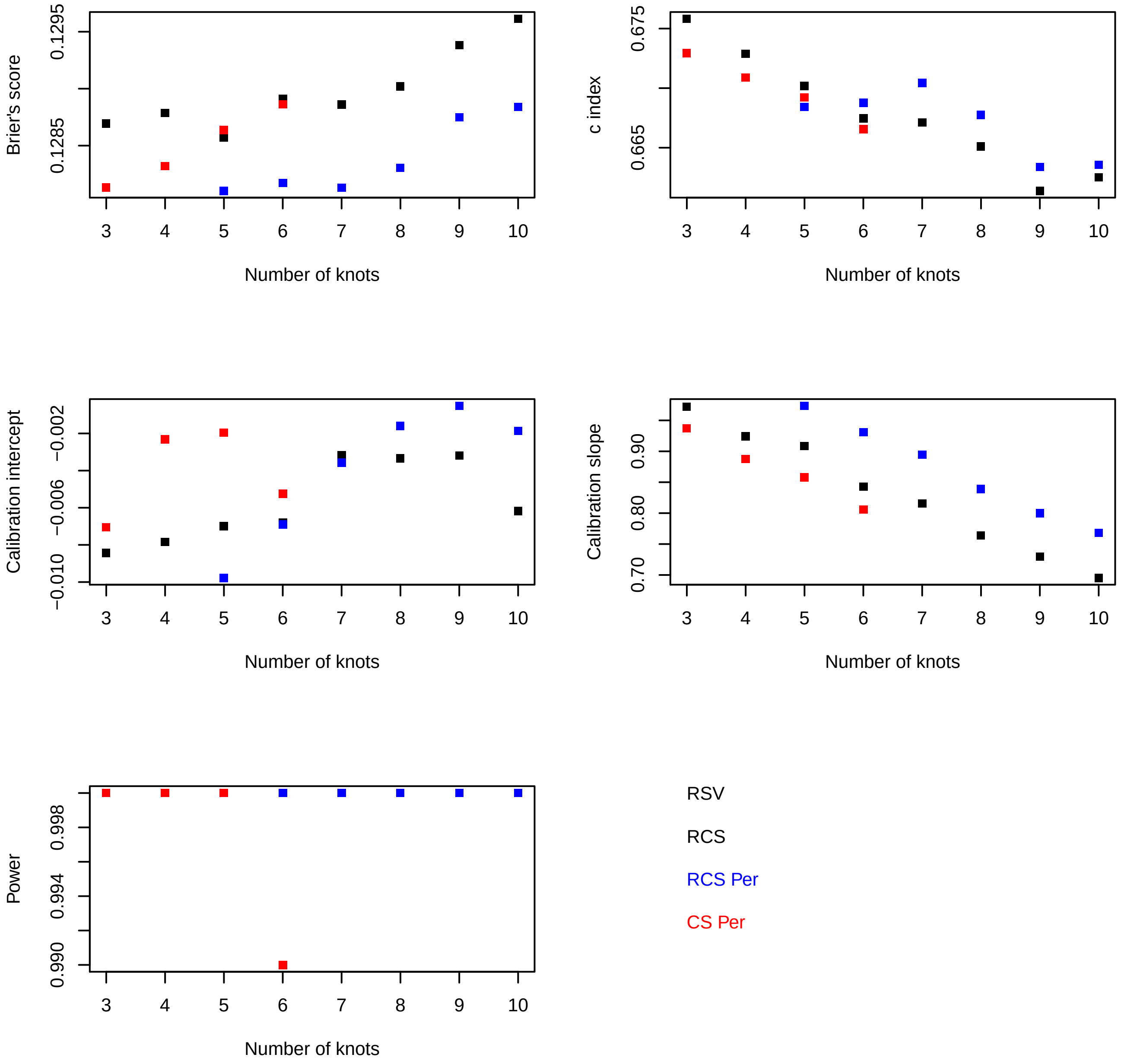
Brier's score
0.1285
0.1295
c index
0.665
0.675
Calibration intercept
−0.010
−0.006
−0.002
Calibration slope
0.70
0.80
0.90
Power
0.990
0.994
0.998
3
4
5
6
7
8
9
10
Number of knots
RSV
RCS
RCS Per
CS Per

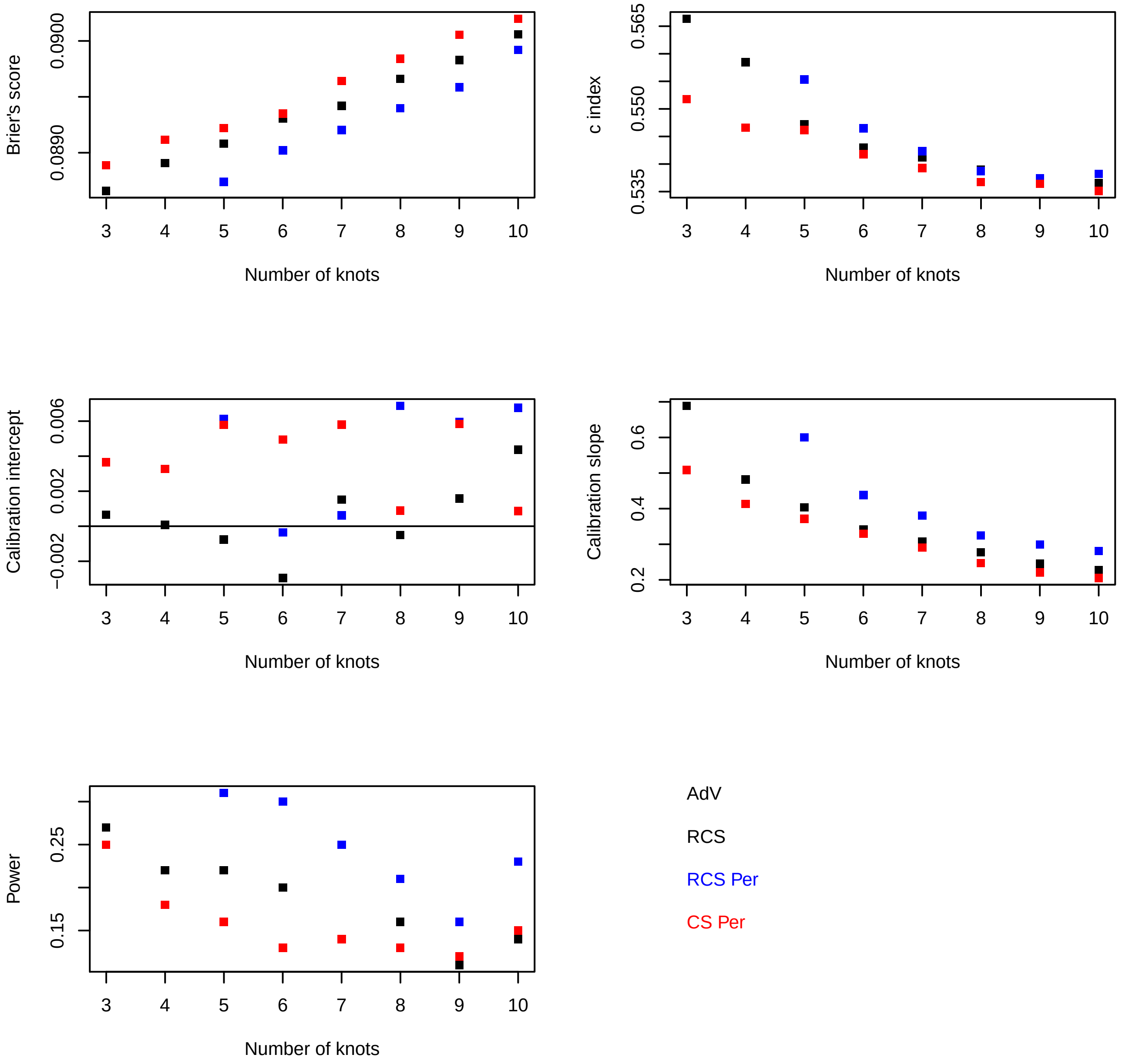
Brier's score
0.0890
0.0900
Number of knots
c index
0.535
0.550
0.565
Calibration intercept
−0.002
0.002
0.006
Calibration slope
0.2
0.4
0.6
Power
0.15
0.25
3
4
5
6
7
8
9
10
AdV
RCS
RCS Per
CS Per

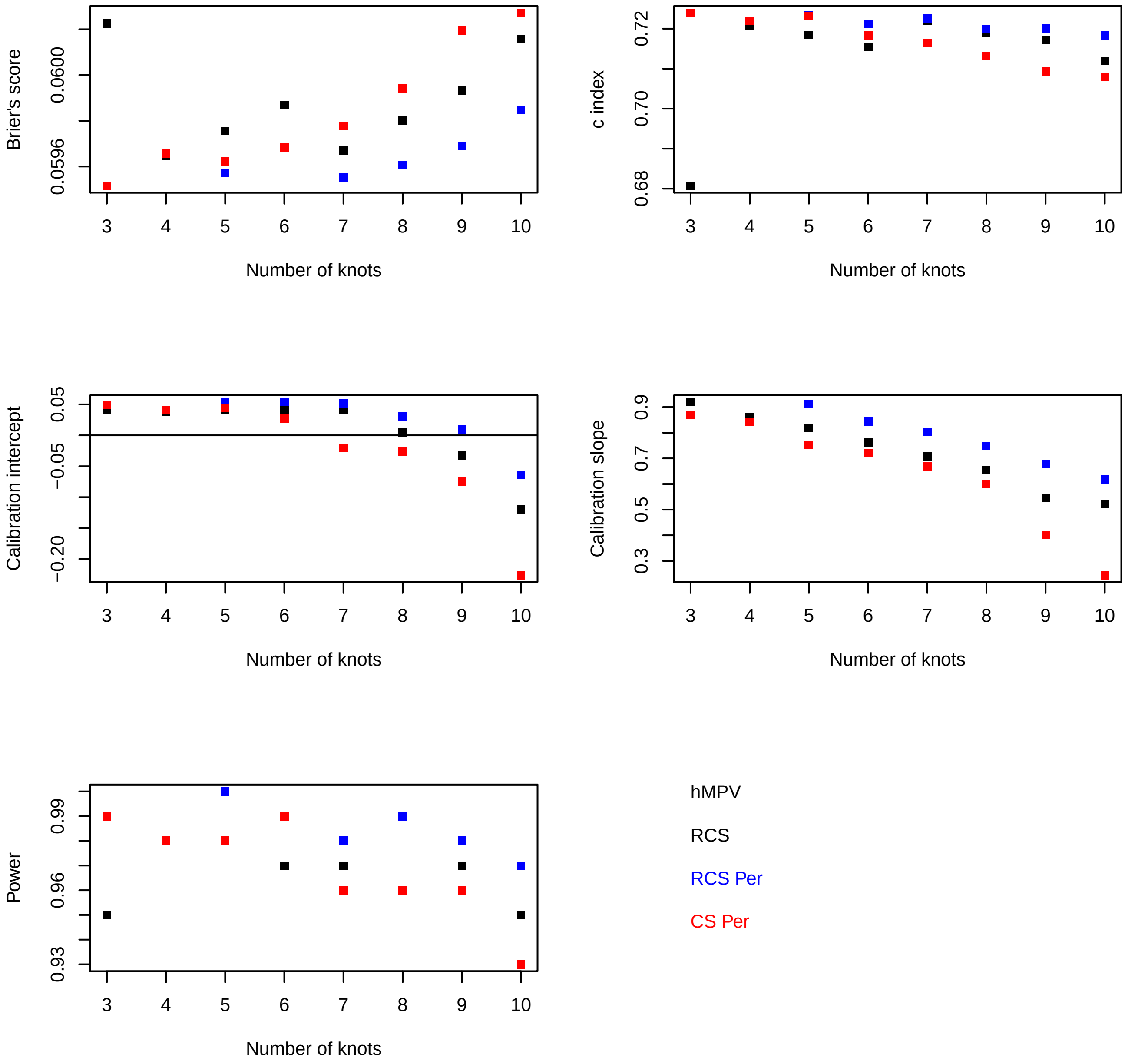

Brier's score
0.0596
0.0600
c index
0.68
0.70
0.72
Calibration intercept
−0.20
−0.05
0.05
Calibration slope
0.3
0.5
0.7
0.9
Power
0.93
0.96
0.99
3
4
5
6
7
8
9
10
Number of knots
hMPV
RCS
RCS Per
CS Per

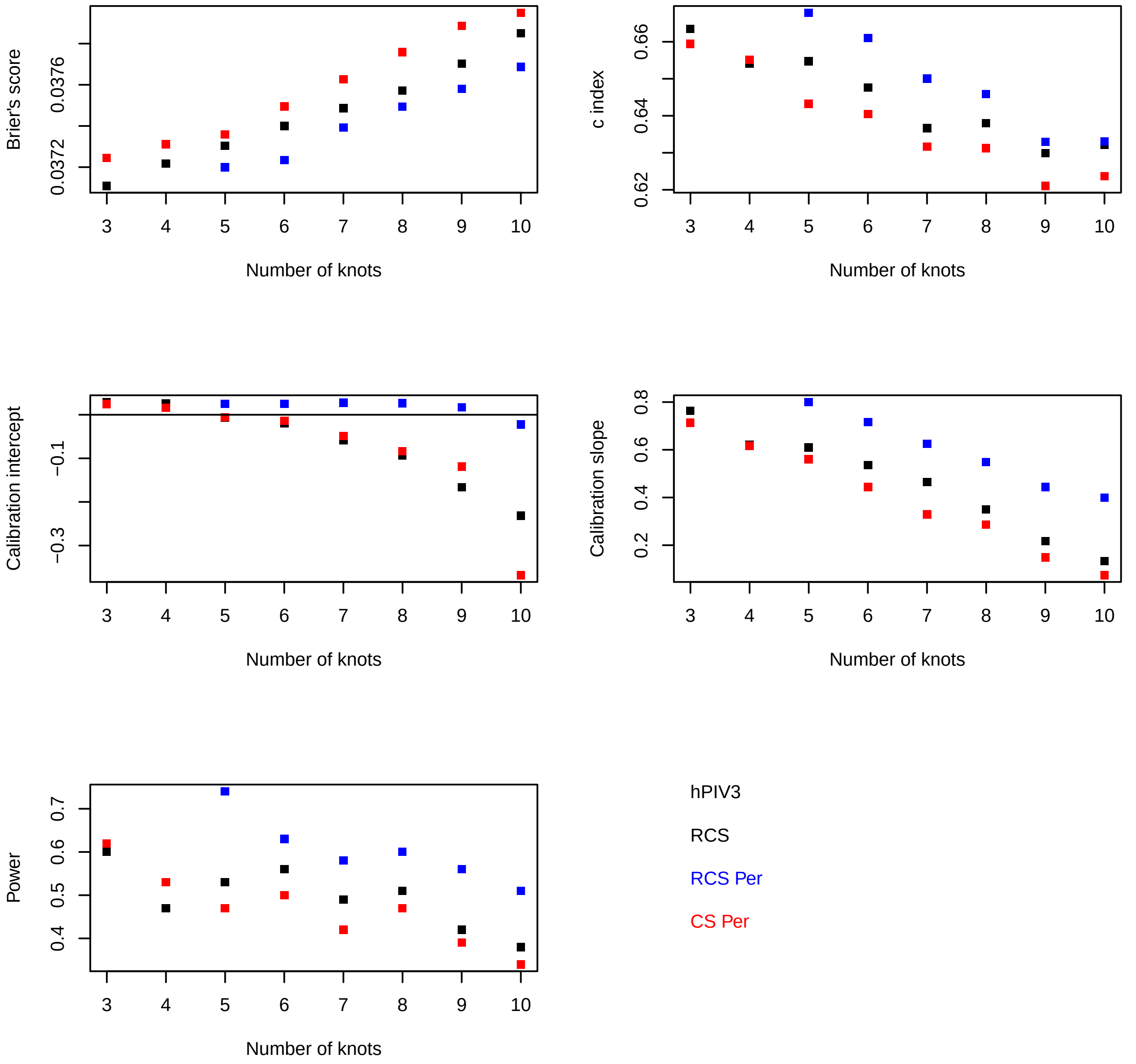
Brier's score
0.0372
0.0376
c index
0.62
0.64
0.66
Calibration intercept
−0.3
−0.1
Calibration slope
0.2
0.4
0.6
0.8
Power
0.4
0.5
0.6
0.7
3
4
5
6
7
8
9
10
Number of knots
hPIV3
RCS
RCS Per
CS Per

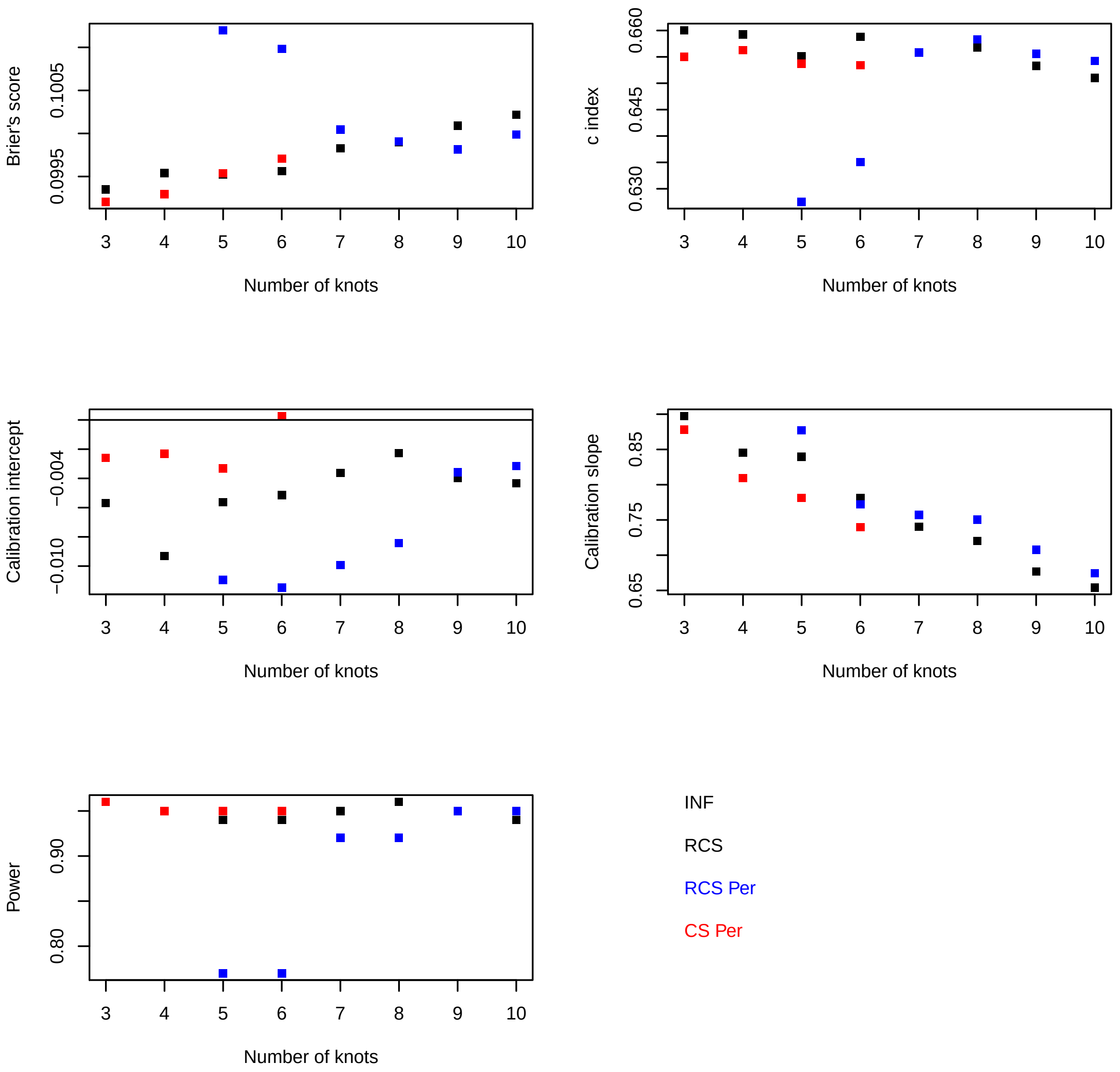
Brier's score
0.0995
0.1005
c index
0.630
0.645
0.660
Calibration intercept
−0.010
−0.004
Calibration slope
0.65
0.75
0.85
Power
0.80
0.90
Number of knots
3 4 5 6 7 8 9 10
INF
RCS
RCS Per
CS Per
