# Supplementary material for: Restricted cubic splines for modelling periodic data
Source: PLoS One. 2020 Oct 28;15(10):e0241364. doi: 10.1371/journal.pone.0241364 (PMC7592770; doi:10.1371/journal.pone.0241364)
Supplement: S2 File — Estimated probabilities obtained from analyses that use different starting points for the periods; the analyses are based on the complete data set of Horton et al. and on the repeated analysis of 500 units. (PDF) [file pone.0241364.s007.pdf]

Additional file 5

Figure 1, page 2-3: superimposed curves of estimated probabilities from 14 models for periodic RCS and periodic CS, which use different period starting points (week 1, 4, 8, 12, ..., 52) and 7 knots. The complete data set of Horton et al. is used. The points on the curves indicate the time point used aas starting point of the period.

Figure 2, page 4-5: superimposed curves of estimated probabilities from 14 models for periodic RCS and periodic CS, which use different period starting points (week 1, 4, 8, 12, ..., 52) and 10 knots. The complete data set of Horton et al. is used. The points on the curves indicate the time point used as starting point of the period.

Figure 3, page 6: superimposed curves of estimated probabilities from 14 models for periodic RCS and periodic CS, which use different period starting points (week 1, 4, 8, 12, ..., 52) and 10 knots. The used data are a random subset with 500 units from the data set of Horton et al. is used. The points on the curves indicate the time point used as starting point of the period.

Figure 4, page 7: superimposed curves of estimated probabilities from 14 models for periodic RCS and periodic CS, which use different period starting points (week 1, 4, 8, 12, ..., 52) and 10 knots. The used data are a random subset with 500 units from the data set of Horton et al. is used (different from the one used for Figure 3 and 5). The points on the curves indicate the time point used as starting point of the period.

Figure 5, page 8: superimposed curves of estimated probabilities from 14 models for periodic RCS and periodic CS, which use different period starting points (week 1, 4, 8, 12, ..., 52) and 10 knots. The used data are a random subset with 500 units from the data set of Horton et al. is used (different from the one used for Figure 3 and 4). The points on the curves indicate the time point used as starting point of the period.

Table, page 9: average unit-based standard deviations of the probability estimates obtained from the 14 models that use different starting points of the periods, averaged across units and subsamples. Each subsample included 500 units from the Horton et al. data set and the subsampling was repeated 500 times. RCSp: periodic RCS, CSp: periodic CS

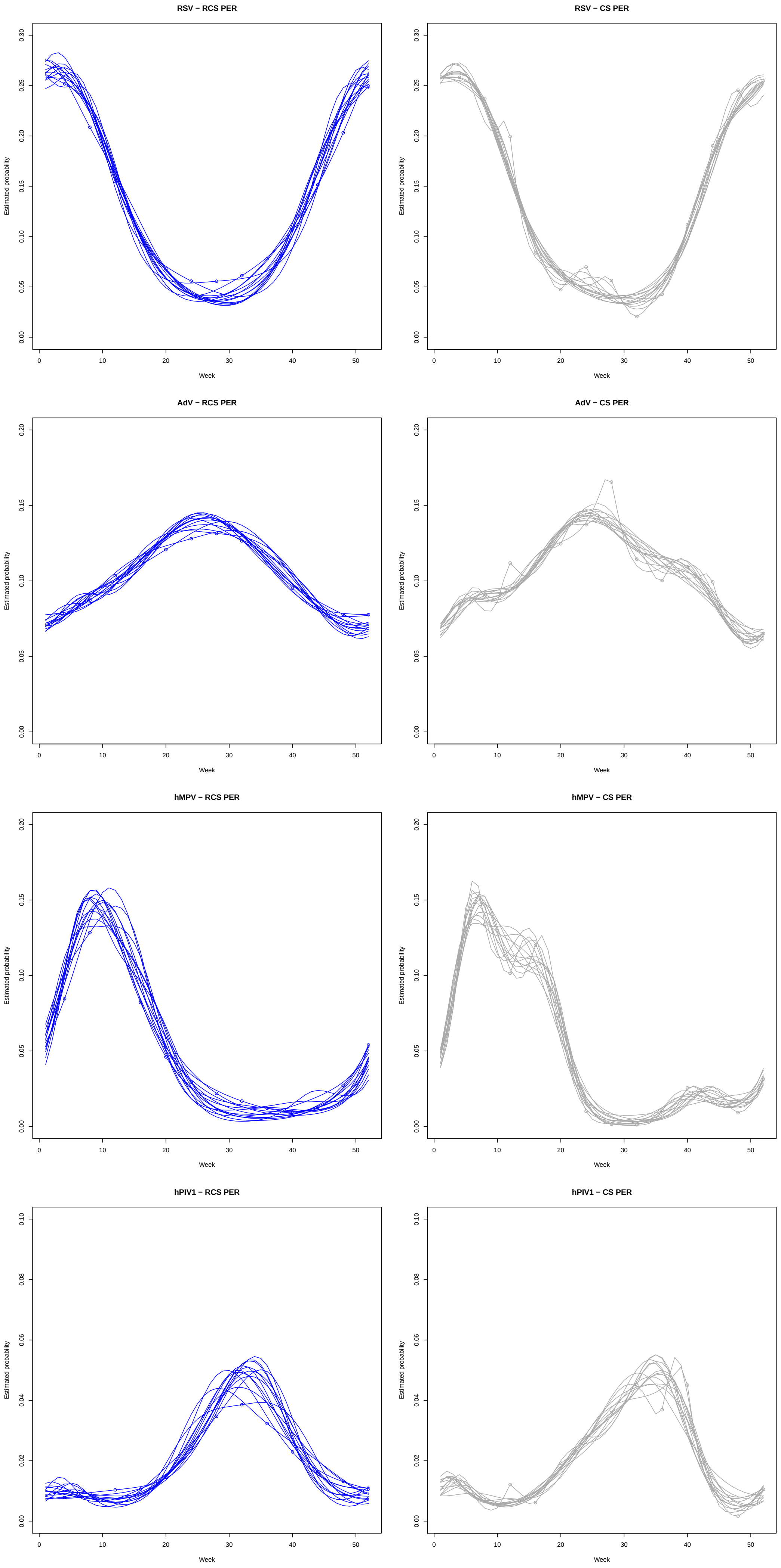
RSV − RCS PER
RSV − CS PER
AdV − RCS PER
AdV − CS PER
hMPV − RCS PER
hMPV − CS PER
hPIV1 − RCS PER
hPIV1 − CS PER
Estimated probability
Week

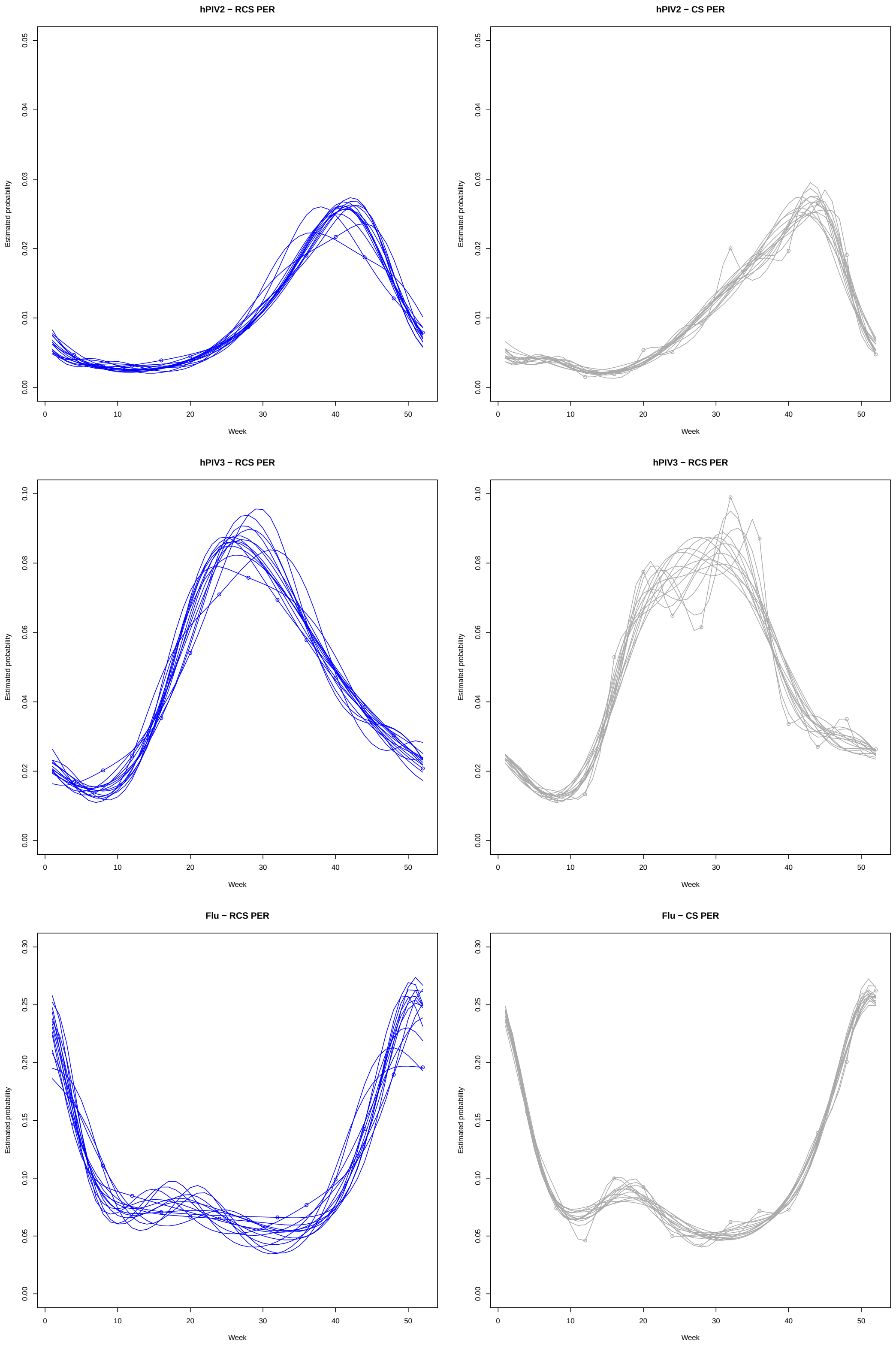

hPIV2 − RCS PER
hPIV2 − CS PER
hPIV3 − RCS PER
hPIV3 − RCS PER
Flu − RCS PER
Flu − CS PER
Estimated probability
Week
0.00 0.01 0.02 0.03 0.04 0.05
0.00 0.02 0.04 0.06 0.08 0.10
0.00 0.05 0.10 0.15 0.20 0.25 0.30
0 10 20 30 40 50

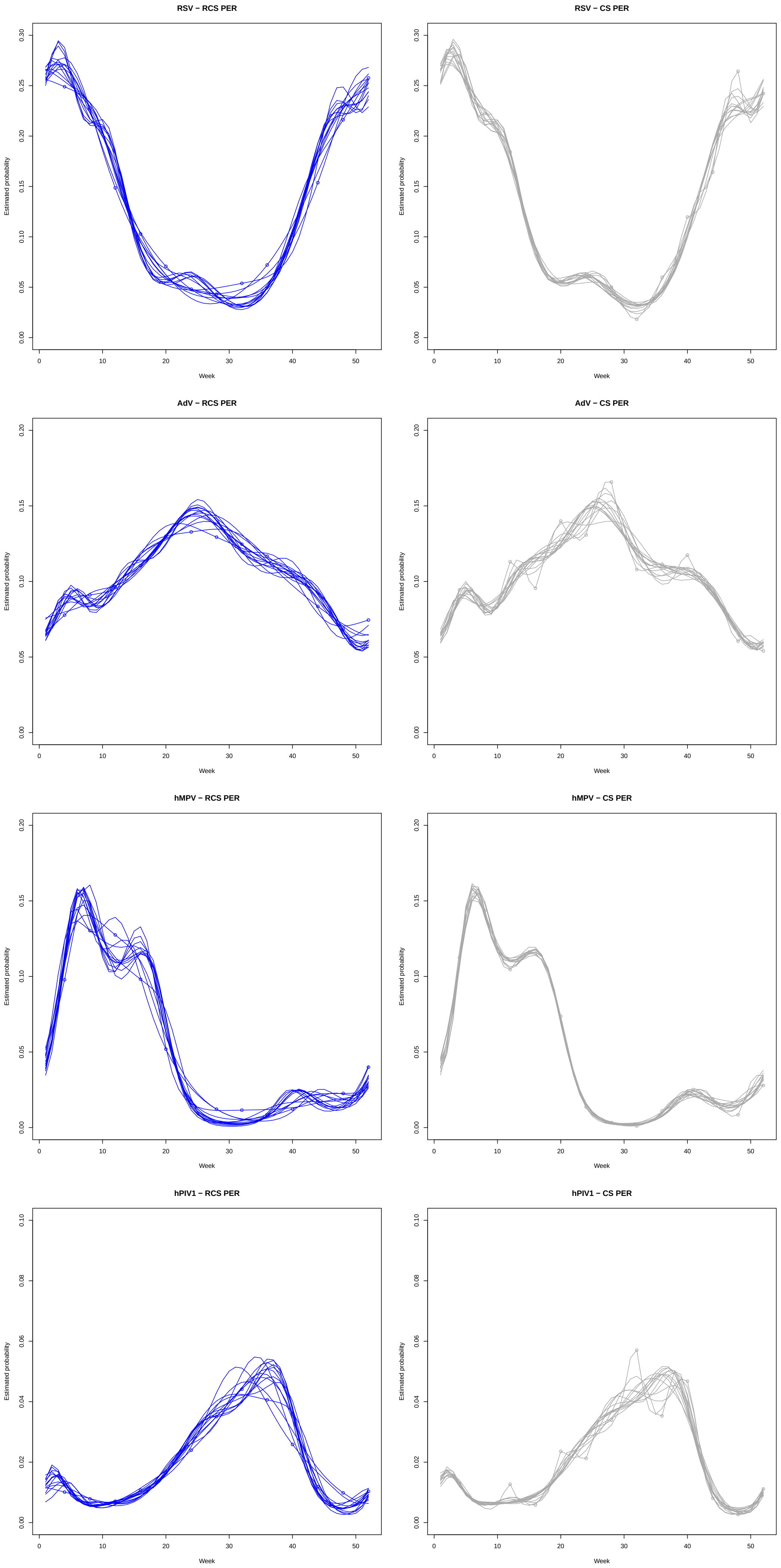
RSV − RCS PER
RSV − CS PER
AdV − RCS PER
AdV − CS PER
hMPV − RCS PER
hMPV − CS PER
hPIV1 − RCS PER
hPIV1 − CS PER
Estimated probability
Week

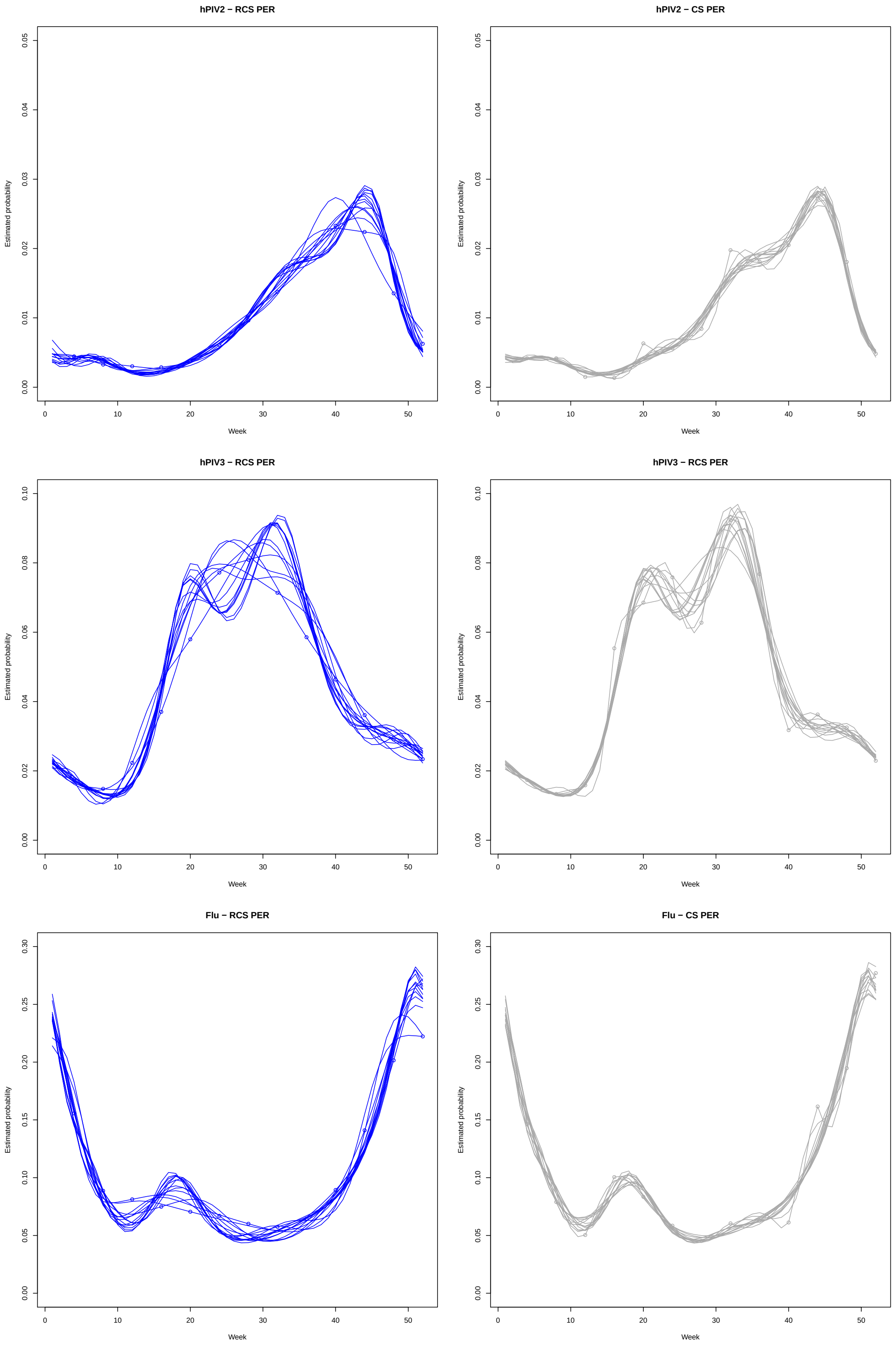
hPIV2 − RCS PER
hPIV2 − CS PER
hPIV3 − RCS PER
hPIV3 − RCS PER
Flu − RCS PER
Flu − CS PER
Estimated probability
Week
0
10
20
30
40
50
0.00
0.01
0.02
0.03
0.04
0.05
0.06
0.08
0.10
0.15
0.20
0.25
0.30

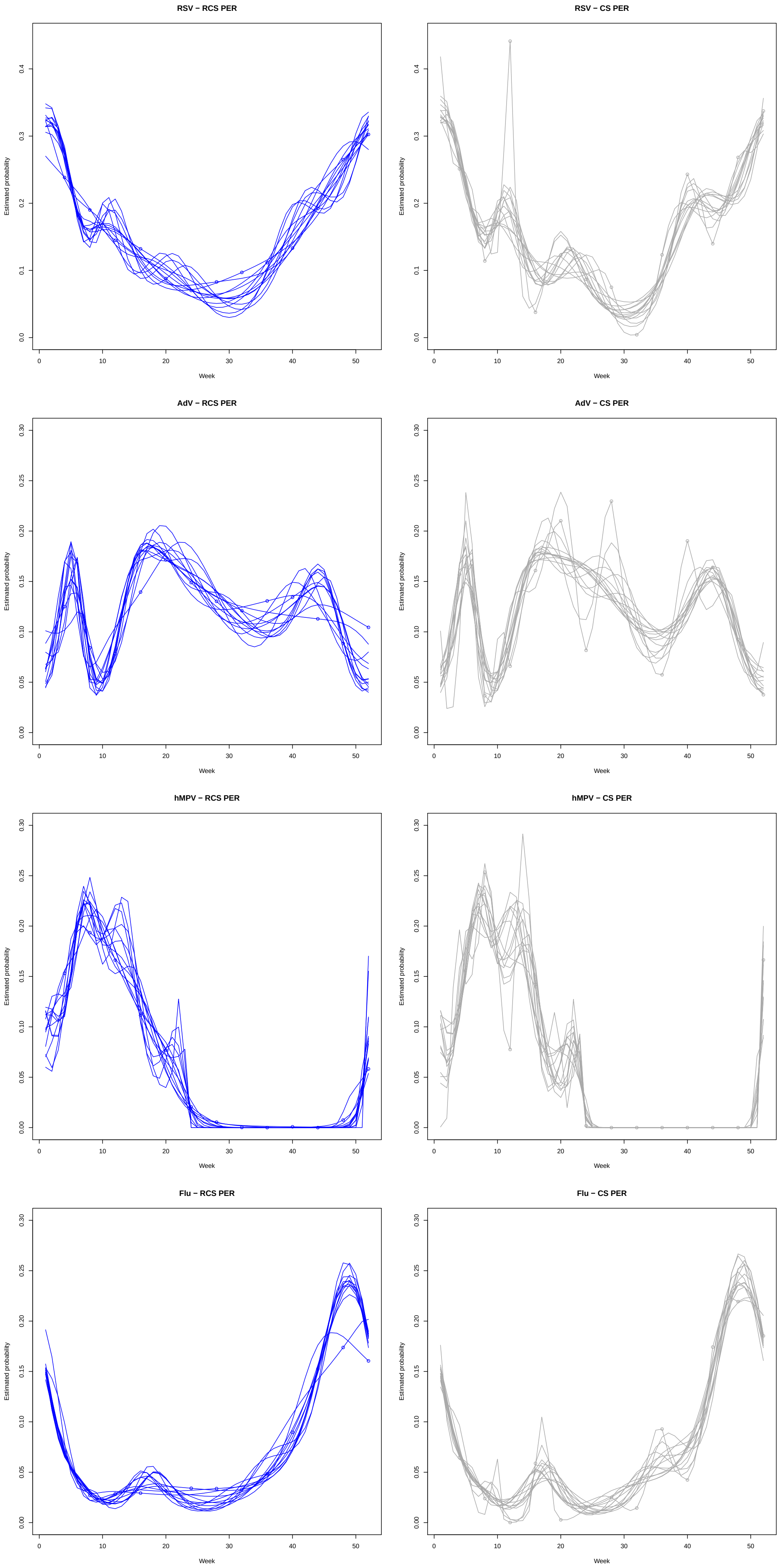
RSV − RCS PER
RSV − CS PER
AdV − RCS PER
AdV − CS PER
hMPV − RCS PER
hMPV − CS PER
Flu − RCS PER
Flu − CS PER
Estimated probability
Week

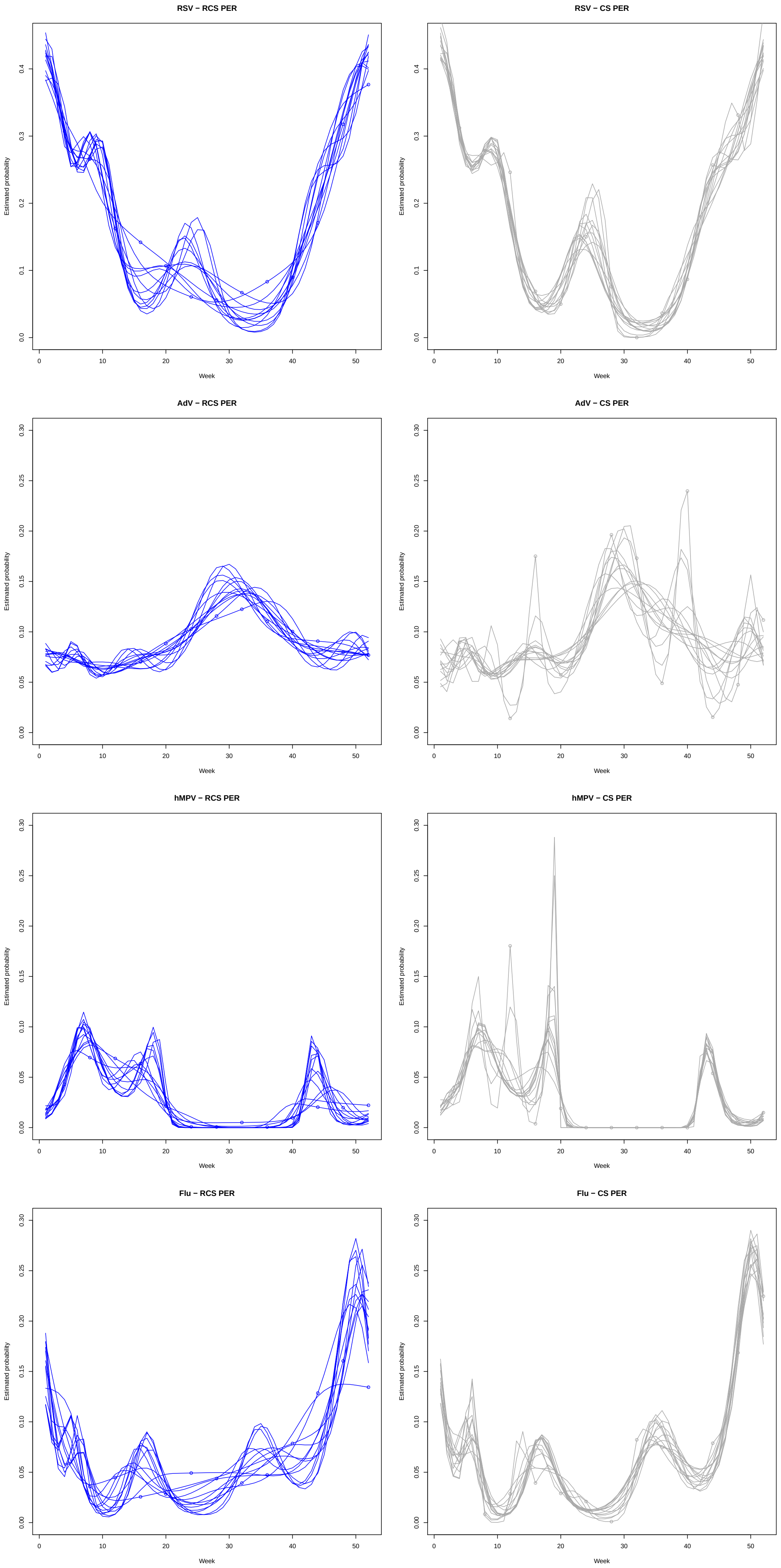
RSV − RCS PER
RSV − CS PER
AdV − RCS PER
AdV − CS PER
hMPV − RCS PER
hMPV − CS PER
Flu − RCS PER
Flu − CS PER
Estimated probability
Week
0.0 0.1 0.2 0.3 0.4
0.00 0.05 0.10 0.15 0.20 0.25 0.30
0 10 20 30 40 50

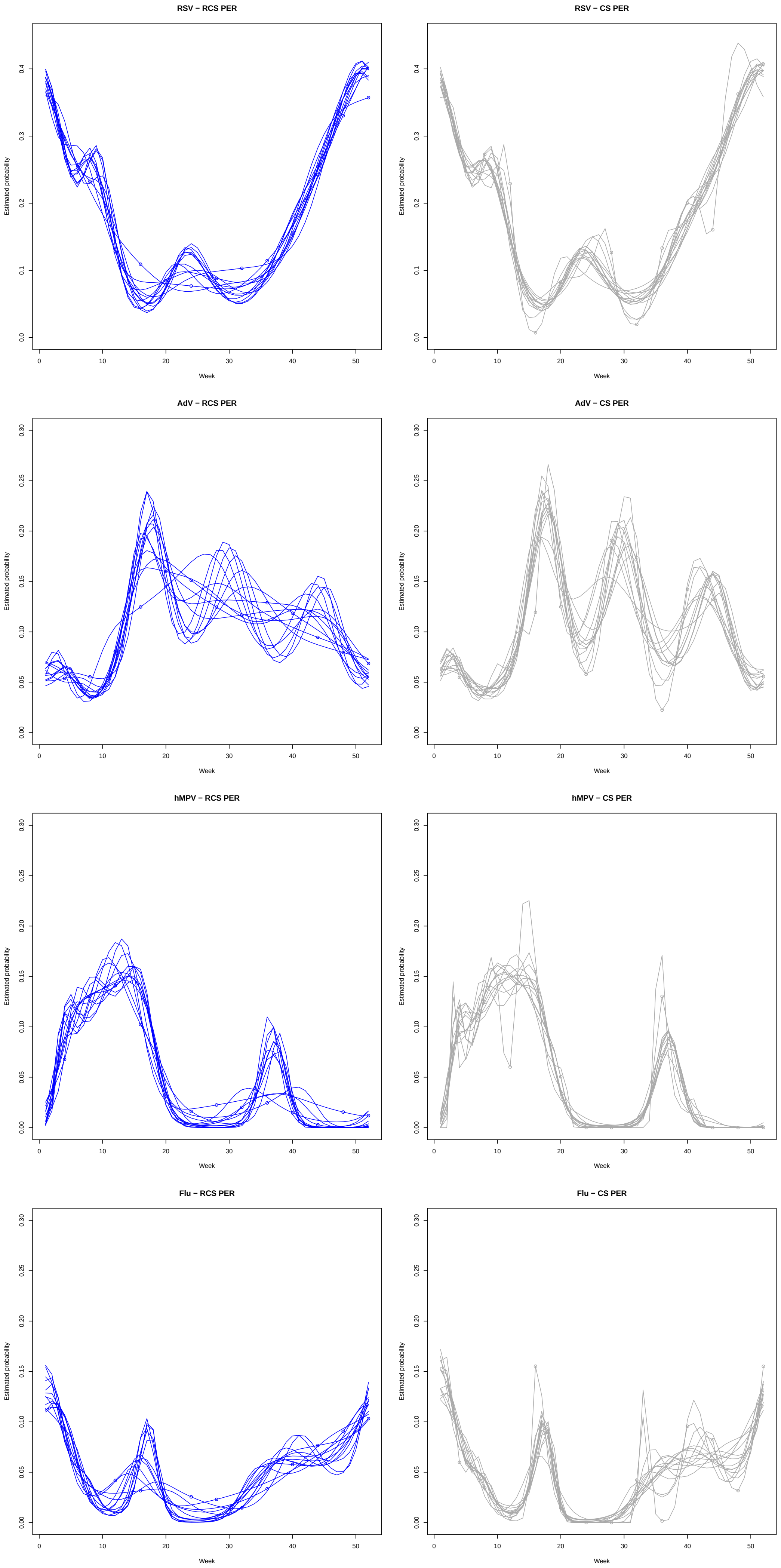
RSV − RCS PER
RSV − CS PER
AdV − RCS PER
AdV − CS PER
hMPV − RCS PER
hMPV − CS PER
Flu − RCS PER
Flu − CS PER
Estimated probability
Week

Knots	RSV-RCSp	RSV-CSp	AdV-RCSp	AdV-CSp	hMPV-RCSp	hMPV-CSp	INF-RCSp	INF-CSp
5	0.023	0.016	0.008	0.012	0.011	0.011	0.009	0.010
6	0.024	0.016	0.011	0.012	0.012	0.010	0.011	0.010
7	0.017	0.018	0.010	0.013	0.009	0.011	0.009	0.011
8	0.019	0.019	0.012	0.013	0.012	0.011	0.010	0.011
9	0.019	0.019	0.013	0.013	0.011	0.013	0.010	0.011
10	0.020	0.018	0.014	0.014	0.012	0.018	0.011	0.012
