# Supplementary material for: Restricted cubic splines for modelling periodic data
Source: PLoS One. 2020 Oct 28;15(10):e0241364. doi: 10.1371/journal.pone.0241364 (PMC7592770; doi:10.1371/journal.pone.0241364)
Supplement: S1 Appendix — (PDF) [file pone.0241364.s008.pdf]

Appendix: Derivation of restricted periodic cubic splines

We present the derivation of a restricted periodic cubic spline that can be used to model periodic data, using the constraints that the value at the beginning and at the end of the period is equal and that the function is smooth also at the beggining/end of the period. First we introduce the parametrization of the restricted cubic splines.

Definition of unrestricted and restricted cubic splines

An unconstrained cubic spline with k knots $t_1 < t_2 < ... < t_k$ is defined as

$$s(x) = \gamma_0 + \gamma_1 x + \gamma_2 x^2 + \gamma_3 x^3 + \sum_{j=1}^{k} \beta_j (x - t_j)^3_+,$$

where $(z)_+$ is equal to z for $z > 0$, 0 otherwise. This definition guarantees that the function is cubic in each interval delimited by the knots and that it is smooth in each knot, the first two derivatives being equal by definition.

When cubic splines are used in a regression model to flexibly model a numerical covariate, $k + 3$ regression parameters are estimated; the part of the design matrix relative to x comprises $k + 3$ columns that contain, respectively, the value of x, its square, its cube and the k transformed cubic truncated functions, whose value depend on the k chosen knots. The estimated coefficients themselves are of little direct interest as they are difficult to interpret, but they can be used to derive the estimated shape of the curve that estimates the association between the numerical covariate and the outcome and its confidence intervals. Moreover, standard statistical testing methods can be used to assess if there is a statistically significant association between the covariate and the outcome variable (testing the null hypothesis $\gamma_1 = \gamma_2 = \gamma_3 = \beta_1 = ... = \beta_k = 0$); Also, the non-linear contribution of the variable can be assessed comparing the nested models that include the cubic spline or only the linear term.

A restricted cubic spline is defined imposing the constraint of linearity in the extremes of the curve (for values $x \leq t_1$ and $x \geq t_k$).

Linearity for $x \leq t_1$ implies that $\gamma_2 = \gamma_3 = 0$, while linearity for $x \geq t_k$ also implies that the spline can be defined using $k - 1$ coefficients, re-parametrizing the function (for example, see the derivation in [23]).

$$s(x) = \gamma_0 + \gamma_1 x + \sum_{j=1}^{k-2} \beta_j \left((x - t_j)^3_+ + (x - t_{k-1})^3_+ \frac{t_k - t_j}{t_k - t_{k-1}} + (x - t_k)^3_+ \frac{t_{k-1} - t_j}{t_k - t_{k-1}} \right).$$

Let us define $b(x, t_j) = \left((x - t_j)^3_+ + (x - t_{k-1})^3_+ \frac{t_k - t_j}{t_k - t_{k-1}} + (x - t_k)^3_+ \frac{t_{k-1} - t_j}{t_k - t_{k-1}} \right)$

$$s(x) = \gamma_0 + \gamma_1 x + \sum_{j=1}^{k-2} \beta_j b(x, t_j).$$

Similarly as described for the unrestricted cubic splines, the part of the design matrix relative to the covariate x will include $k - 1$ columns, including a linear term and $k - 2$ additional terms, defined by the transformations of the covariate given by the $b()$ basis functions.

Derivation of a periodic restricted cubic spline

We define a periodic restricted cubic spline as a restricted periodic spline with two additional constraints: (i) the value at the beginning (x_{min}) and at the end of the period (x_{max}) are equal (s(x_{min})=s(x_{max})) and (ii) the function is smooth at the extremes (the equality of the first derivatives $s'(x_{min}) = s'(x_{max})$ is a sufficient condition, as by definition the function is linear in the extremes and the second derivatives are therefore equal to zero). Note that x_{min} and x_{max} denote the beginning and end of the period and do not need to be attained in the data.

The constraint s(x_{min})=s(x_{max}) implies that

$$\gamma_0 + \gamma_1 x_{min} = \gamma_0 + \gamma_1 x_{max} + \sum_{j=1}^{k-2} \beta_j b(x_{max}, t_j),$$

and consequently that

$$\beta_{k-2} = \frac{\gamma_1(x_{min} - x_{max}) - \sum_{j=1}^{k-3} \beta_j b(x_{max}, t_j)}{b(x_{max}, t_{k-2})}$$

and that

$$s(x) = \gamma_0 + \gamma_1 \left(x + \frac{x_{min} - x_{max}}{b(x_{max}, t_{k-2})} b(x, t_{k-2}) \right) + \sum_{j=1}^{k-3} \beta_j \left(b(x, t_j) - \frac{b(x_{max}, t_j)}{b(x_{max}, t_{k-2})} b(x, t_{k-2}) \right) \tag{1}$$

Deriving the first derivatives of the $s()$ function in the extremes we obtain

$$s'(x_{min}) = \gamma_1 \text{ and}$$

$$\begin{aligned} s'(x_{max}) =& \gamma_1 \left(1 + \frac{x_{min} - x_{max}}{b(x_{max}, t_{k-2})} b'(x_{max}, t_{k-2}) \right) + \\ &+ \sum_{j=1}^{k-3} \beta_j \left(b'(x_{max}, t_j) - \frac{b(x_{max}, t_j)}{b(x_{max}, t_{k-2})} b'(x_{max}, t_{k-2}) \right). \end{aligned}$$

Imposing their equality to guarantee the smoothness of the function we derive the constrained value of an additional regression coefficient

$$\beta_{k-3} \left(b'(x_{max}, t_{k-3}) - \frac{b(x_{max}, t_{k-3})}{b(x_{max}, t_{k-2})} b'(x_{max}, t_{k-2}) \right)$$

$$= -\gamma_1 \left(\frac{x_{min} - x_{max}}{b(x_{max}, t_{k-2})} b'(x_{max}, t_{k-2}) \right) - \sum_{j=1}^{k-4} \beta_j \left(b'(x_{max}, t_j) - \frac{b(x_{max}, t_j)}{b(x_{max}, t_{k-2})} b'(x_{max}, t_{k-2}) \right)$$

$$\beta_{k-3} = \frac{-\gamma_1 \left(\frac{x_{min} - x_{max}}{b(x_{max}, t_{k-2})} b'(x_{max}, t_{k-2}) \right) - \sum_{j=1}^{k-4} \beta_j \left(b'(x_{max}, t_j) - \frac{b(x_{max}, t_j)}{b(x_{max}, t_{k-2})} b'(x_{max}, t_{k-2}) \right)}{b'(x_{max}, t_{k-3}) - \frac{b(x_{max}, t_{k-3})}{b(x_{max}, t_{k-2})} b'(x_{max}, t_{k-2})}.$$

Substituting in the equation 1

$$
\begin{aligned}
s(x) =& \gamma_0 + \gamma_1 \left(x + \frac{x_{min} - x_{max}}{b(x_{max}, t_{k-2})} b(x, t_{k-2}) \right) + \\
& \sum_{j=1}^{k-4} \beta_j \left(b(x, t_j) - \frac{b(x_{max}, t_j)}{b(x_{max}, t_{k-2})} b(x, t_{k-2}) \right) + \\
& \beta_{k-3} \left(b(x, t_{k-3}) - \frac{b(x_{max}, t_{k-3})}{b(x_{max}, t_{k-2})} b(x, t_{k-2}) \right) \\
=& \gamma_0 + \gamma_1 \left(x + \frac{x_{min} - x_{max}}{b(x_{max}, t_{k-2})} b(x, t_{k-2}) \right) + \\
& \sum_{j=1}^{k-4} \beta_j \left(b(x, t_j) - \frac{b(x_{max}, t_j)}{b(x_{max}, t_{k-2})} b(x, t_{k-2}) \right) + \\
& - \gamma_1 \frac{\frac{x_{min} - x_{max}}{b(x_{max}, t_{k-2})} b'(x_{max}, t_{k-2})}{b'(x_{max}, t_{k-3}) - \frac{b(x_{max}, t_{k-3})}{b(x_{max}, t_{k-2})} b'(x_{max}, t_{k-2})} \left(b(x, t_{k-3}) - \frac{b(x_{max}, t_{k-3})}{b(x_{max}, t_{k-2})} b(x, t_{k-2}) \right) + \\
& - \frac{\sum_{j=1}^{k-4} \beta_j \left(b'(x_{max}, t_j) - \frac{b(x_{max}, t_j)}{b(x_{max}, t_{k-2})} b'(x_{max}, t_{k-2}) \right)}{b'(x_{max}, t_{k-3}) - \frac{b(x_{max}, t_{k-3})}{b(x_{max}, t_{k-2})} b'(x_{max}, t_{k-2})} \left(b(x, t_{k-3}) - \frac{b(x_{max}, t_{k-3})}{b(x_{max}, t_{k-2})} b(x, t_{k-2}) \right) \\
=& \gamma_0 + \\
& \gamma_1 \left(x + \frac{x_{min} - x_{max}}{b(x_{max}, t_{k-2})} b(x, t_{k-2}) \right. \\
& \left. - \frac{\frac{x_{min} - x_{max}}{b(x_{max}, t_{k-2})} b'(x_{max}, t_{k-2})}{b'(x_{max}, t_{k-3}) - \frac{b(x_{max}, t_{k-3})}{b(x_{max}, t_{k-2})} b'(x_{max}, t_{k-2})} \left(b(x, t_{k-3}) - \frac{b(x_{max}, t_{k-3})}{b(x_{max}, t_{k-2})} b(x, t_{k-2}) \right) \right) + \\
& \sum_{j=1}^{k-4} \beta_j \left(b(x, t_j) - \frac{b(x_{max}, t_j)}{b(x_{max}, t_{k-2})} b(x, t_{k-2}) + \right. \\
& \left. - \frac{b'(x_{max}, t_j) - \frac{b(x_{max}, t_j)}{b(x_{max}, t_{k-2})} b'(x_{max}, t_{k-2})}{b'(x_{max}, t_{k-3}) - \frac{b(x_{max}, t_{k-3})}{b(x_{max}, t_{k-2})} b'(x_{max}, t_{k-2})} \left(b(x, t_{k-3}) - \frac{b(x_{max}, t_{k-3})}{b(x_{max}, t_{k-2})} b(x, t_{k-2}) \right) \right)
\end{aligned} \tag{2}
$$

we derived the spline function that uses $k-3$ regression parameters to model the non-linear periodic association between the numerical covariate and the outcome. Note that the periodic version of the RCS does not include a separate term for the linear component of x, and an overall test for the non-linear period association of the covariate x with the outcome can be based on the null hypothesis: $\gamma_1 = \beta_1 = ... = \beta_{k-4} = 0$.

Derivation of a periodic cubic spline

Zhang et al. [6] proposed the use of periodic cubic spline functions to model periodic data, using the same two additional constraints that we propose: the equality of the function and its smoothness in the extremes. Using an unconstrained CS, the smoothness in the extremes implies the equality of the first two derivatives and, overall, k regression parameters are used.

Zhang et al. [6] derived the parametrization assuming that the period was defined between 0 and T, while here we present the periodic CS using the same notation introduced for the periodic RCS. The spline function is defined as

$$s(x) = \gamma_0 + \sum_{j=1}^{k} \beta_j s_j(x)$$

$$s_j(x) = a_j x + b_j x^2 + c_j x^3 + (x - t_j)^3_+$$

with

$$a_j = -\frac{1}{x_{max} - x_{min}} \left(\frac{x_{max}^2 + x_{min}^2 + 4x_{max}x_{min}}{2}(x_{max} - t_j) - \frac{3(x_{max} + x_{min})}{2}(x_{max} - t_j)^2 + (x_{max} - t_j)^3 \right),$$

$$b_j = \frac{3(x_{max} + x_{min})(x_{max} - t_j)}{2(x_{max} - x_{min})} - \frac{3(x_{max} - t_j)^2}{2(x_{max} - x_{min})},$$

$$c_j = -\frac{(x_{max} - t_j)}{x_{max} - x_{min}}.$$

Assuming that the period of x is defined on $[0, T]$ (i.e., $x_{min} = 0, x_{max} = T$), a_j, b_j and c_j simplify to

$$a_j = -\frac{T(T - t_j)}{2} + \frac{3(T - t_j)^2}{2} - \frac{(T - t_j)^3}{T},$$

$$b_j = \frac{3(T - t_j)}{2} - \frac{3(T - t_j)^2}{2T},$$

$$c_j = -\frac{T - t_j}{T},$$

which are the same values reported in the original publication, with the exception of c_j that was reported without the minus sing in the original paper. This should be regarded as a typo, as we verified that the equations produce a periodic CS using the formulas reported above.

Appendix: Using the peRiodiCS R package

The functions and examples described in this paper are included in an R package, which can be used from the users that have the R statistical environment installed; the open source installation files for the R program and installation instructions can be found in [13]. We suggest that the users download and install the most recent and stable versions of R. The peRiodiCS package [12] is available on CRAN and can be installed entering the following command in the R console window:

```
install.packages("peRiodiCS")
```

The package functions can be used after loading the package with :

```
library("peRiodiCS")
```

The sample dataset of Horton and colleagues reanalyzed in this paper [2] is included in the package. Data and info about it can be loaded with the following commands:

```
# load dataset
data("viral_east_mediteranean")
# show help explaining the dataset
help("viral_east_mediteranean")
```

(note that the # character at the beginning of a line precedes a comment, which is not executed).

The logistic regression models fitted in the following examples use `RSV` positivity as the outcome and the `EpiWeek` variable as a periodic explanatory variable, which represents the week of the year when the measurement was made and ranges from 1 to 53.

In general, the explanatory variable needs to be expressed in units within the period. For example, if the period is the calendar year and the variable is measured over a series of years, the day in the year can be used, giving the value of 1 to 1st of January, 2 to 2nd of January, up to day 365. If the period is a day, the periodic variable can be defined as the number of minutes from midnight.

The logistic regression model using periodic RCS with 5 knots is fitted with the following command:

```
# model viral infections vs weeks
model <- glm(RSV ~ rcs_per(EpiWeek, nk = 5),
                          data = viral_east_mediteranean,
                          family = "binomial")
```

A different number of knots can be specified by changing the value of the `nk` parameter. The locations of the knots are determined in the same way as in the `rms` R package [15] for restricted cubic splines - by using the `rcspline.eval` function from the `Hmisc` package [24] in the background. For this example the knots are positioned at weeks 2, 10, 22, 38 and 51.

The knot locations can be retrieved by using either one of the following functions, which return only the locations:

```
# retrieve the location of knots used and assign them to Knots variable
Knots <- Hmisc::rcspline.eval(x = viral_east_mediteranean$EpiWeek,
                              nk = 5, knots.only = TRUE)

# extract knot locations from attributes of the model
attr(rcs_per(viral_east_mediteranean$EpiWeek, nk = 5), "knots")
```

The estimated splines depend on the location of the knots. If these locations are not supplied by the user, they are determined from the distribution of data used in the model. If the user wishes to use the fitted model to obtain predictions on new data, the same knots used to fit the original models need to be used also on the new data.

The knots for a spline can be placed manually at values motivated by specific knowledge, for example when changes in the outcome are expected at specific values of the explanatory variable. If the user wishes to specify the knot locations and fit a new model, she can do so using the following commands:

```
# specify own knots
Knots2 <- c(5, 10, 20, 35, 50)
model2 <- glm(RSV ~ rcs_per(EpiWeek, knots = Knots2),
```

```
                                 data = viral_east_mediteranean,
                                 family = "binomial")
```

The score test can be used to test if there is an overall association between the week and the outcome:

```
anova(model, test="Rao")
```

The score test indicates a strong statistically significant association between week and RSV positivity ($P < 0.0001$).

The overall summary of the logistic regression model is given using the:

```
summary(model)
```

The output provides the values of the two estimated regression coefficients for the periodic RCS, which are difficult to interpret. Instead, the estimated probabilities of RSV positivity as a function of the week, with their 95% CI, can be plotted as a smooth curve using a function from the package:

```
# plot model (with many points, to make it smooth)
plot_per_mod(Model = model, XvarName = "EpiWeek", Smooth = TRUE)
```

Similarly, models using periodic CS or regular RCS can be fitted using, respectively, the `cs_per()` or `rcs()` functions instead of the `rcs_per()` function.

The user can also generate and export only the design matrix (the basis functions) that is used in estimating the model, if she wishes to use a different software for the statistical analyses. The following code generates the values of the basis functions for RCS, periodic RCS and periodic CS and saves them into an R object.

```
# RCS - includes the x term
design_matrix_rcs <-
    Hmisc::rcspline.eval(viral_east_mediteranean$EpiWeek,
    knots = Knots, inclx = TRUE)

# periodic RCS
design_matrix_rcs_per <- rcs_per(viral_east_mediteranean$EpiWeek,
                                 knots = Knots)

# periodic CS
design_matrix_cs_per <- cs_per(viral_east_mediteranean$EpiWeek,
                                 knots = Knots)
```

The design matrix can be saved to a comma separated values file (CSV):

```
# write RCS design matrix to CSV file
write.csv2(design_matrix_rcs,
            file = "design_matrix_rcs.csv",
            row.names = FALSE)
```

Example code for the use of periodic CS for modelling RSV positivity can be run in R using the command:

```
# periodic CS example model and plot
example(cs_per)
```
